# Preserving the Motif-Relevant Structure in Graph Neural Networks: An Entropy-Aware Study of Aggregation and Depth

**DOI:** 10.3390/e28101043

**Published:** 2026-09-22

**Authors:** Lidija Kunst, Friedhelm Schwenker, Hans A. Kestler

**Affiliations:** 1 Institute of Medical Systems Biology, Ulm University, Albert-Einstein-Allee 11, 89069 Ulm, Germany; 2 Institute of Neural Information Processing, Ulm University, James-Franck-Ring, 89081 Ulm, Germany

**Keywords:** deep learning, graph representation learning, graph neural networks, multilabel classification, network motifs, graphlets, subgraph matching

## Abstract

Graph neural networks have shown strong potential for learning structural representations of biological networks. However, repeated message passing may blur local structural signals that are relevant for motif- and graphlet-based analysis. This paper investigates multilabel graphlet classification in protein–protein interaction networks to test whether GNNs can serve as reliable structural indicators for motif mining. We focus on two central design choices of message-passing neural networks: network depth, which determines the range of propagated neighborhood information, and neighborhood aggregation, which determines how this information is combined. We compare mean, max, and sum aggregation across increasing message-passing depths and across input feature sets with different levels of structural informativeness. Dropout and batch normalization are considered additional architectural factors. The resulting models are evaluated using multilabel classification metrics alongside homophily measures and Jensen–Shannon divergence to analyze how label distribution patterns impact prediction performance. Our approach acknowledges the structural limitations of GNNs but seeks to determine whether they can serve as reliable “hints” for the presence or absence of graphlets. To connect graphlet prediction with motif discovery, we further include a post hoc probability-guided motif search in which predicted graphlet probabilities are used to prioritize candidate regions for deterministic motif detection. This study provides an empirical analysis of how depth, aggregation, and feature informativeness interact in motif-oriented graph representation learning.

## 1. Introduction

Graphs are powerful tools for modeling real-world systems, especially in biology. Driven by growing interest in network biology, extensive cellular networks—such as transcriptional, metabolic, and protein–protein interaction maps—have already been characterized for numerous model organisms, most notably *Escherichia coli* and *Saccharomyces cerevisiae* [1]. In protein–protein interaction (PPI) networks, nodes are proteins, and edges correspond to physical bindings between them, forming what is known as the “interactome” [2,3,4,5,6,7,8]. To reconstruct these interactomes, researchers primarily combine literature-curated data, computational predictions, and systematic experimental techniques like yeast two-hybrid (Y2H) assays and affinity purification mass spectrometry (AP/MS) [8,9,10]. Unlike regulatory networks, the edges in PPIs are undirected, representing mutual, non-causal interactions between protein pairs [1,11]. The local structure of a network around a node can reveal important biological information. These localized wiring patterns are typically quantified by counting how frequently a node participates in so-called graphlets—small induced subgraphs [12]. The related concept of motifs was introduced in 2002 as small subgraphs that occur more frequently than expected in randomized counterparts [3,5,13,14,15,16]. In contrast to graphlet-based approaches that rely on raw frequency vectors, motif analyses build on statistical significance profiles. Each subgraph is assigned a significance score based on its overrepresentation relative to a null model [13,14,17]. Functional modules such as protein complexes and signaling pathways are encoded as subgraphs within the interactome. These modules mediate enzymatic reactions, signal transduction and cellular decision-making [17,18,19]. However, identifying or counting subgraphs within large networks is computationally challenging: subgraph isomorphism is NP-indeterminate, and determining exact frequency becomes intractable as network size increases [20,21,22].

Motif discovery algorithms can be broadly divided into two main categories: subgraph census (exhaustive search) and subgraph sampling (approximate counting) [14,16]. The subgraph census approach aims to determine the exact frequency of all subgraphs of a certain size *k* that occur within the input network *G*. Due to the combinatorial nature of subgraph enumeration and the computational cost of subgraph isomorphism checking, this method becomes infeasible for large graphs. Popular tools implementing exhaustive search include: MAVisto, NeMoFinder and Kavosh [14,16]. Sampling-based algorithms estimate motif frequencies by drawing a large number of random subgraph samples of size *k* from the input network. Sampling methods are often used when motif detection is applied exploratorily or when full enumeration is computationally infeasible. Notable implementations include: MFinder [23] and FANMOD [24]. Analytic motif counting methods aim to estimate motif frequencies mathematically. Two major techniques are matrix-based and decomposition-based counting. Matrix-based methods leverage combinatorial properties to derive linear relationships between subgraphs of different sizes [14,25]. The result is a system of linear equations that can be solved using matrix operations. Tools like ORCA [25] and JESSE [26] use such systems to compute graphlet counts efficiently without the direct enumeration of all instances. Decomposition-based methods break down each motif into structural primitives, such as shared neighbors, wedge patterns, or overlapping triangles. By computing the frequency of these smaller building blocks, the algorithm infers the total count of larger subgraphs. However, for large biological networks, even state-of-the-art tools can require hours or even days to compute motif frequencies [14,25].

While graph neural networks (GNNs) are powerful tools for general node classification, their theoretical and empirical limitations in capturing local topological structures—such as subgraph isomorphism and motif counting—remain a critical challenge [27,28]. The literature suggests that standard message-passing architectures are inherently limited by their equivalence to the Weisfeiler–Lehman (WL) graph isomorphism test, preventing them from reliably distinguishing specific graph patterns or counting induced subgraphs of size three or more [27]. This raises a fundamental question: can GNNs generate distinct embeddings for nodes with different roles in a subgraph to accurately classify their membership, even if exact counting remains theoretically unreachable? To investigate this, we cast motif and graphlet detection as a multilabel node classification problem using GraphSAGE [29]. Our approach acknowledges the structural limitations of GNNs but seeks to determine whether they can serve as reliable “hints” for the presence or absence of graphlets. By using predicted probabilities to guide a rule-based subgraph detector, we bridge the gap between representation learning and deterministic mining, evaluating whether GNNs can provide a useful complement to traditional search. Crucially, we move beyond the coarse homophily–heterophily dichotomy, analyzing the interplay between feature informativeness—ranging from constant scalars to spectral moments—and topological properties.

This work provides a comprehensive empirical investigation into motif-oriented graph representation learning through the following key contributions:

- **Framing Motif Detection as a Multilabel Task:** We utilize isomorphism-dependent downstream tasks to probe the expressiveness limits of message-passing architectures, testing the ability of GNNs to distinguish node roles within larger structures.
- **Systematic Architectural Grid Search:** Through a large-scale grid search of 396 configurations, we isolate the impacts of network depth, aggregation operators, and normalization. We demonstrate that performance degradation in deep models is driven by a complex interplay between feature informativeness and topological convergence, rather than oversmoothing alone.
- **Fine-Grained Topological Analysis:** We analyze how embedding separability and node homophily evolve across neighborhoods, offering a nuanced explanation for performance fluctuations that traditional “oversmoothing” narratives often overlook.
- **Transductive vs. Inductive Generalization:** By evaluating across both settings, we demonstrate that topology-aware feature engineering (such as spectral moments) enables GNNs to learn effectively even from minimal or dummy feature representations.
- **Probability-Guided Hybrid Detectors:** Recognizing that GNNs cannot perform exact subgraph matching, we introduce a post hoc, probability-guided search. This approach leverages GNN outputs to prioritize candidates for rule-based detectors, investigating whether this hybrid pipeline can learn to locate structural patterns efficiently. While our analysis reveals that high label homophily in protein–protein interaction networks limits the reduction in computational complexity, the resulting precision highlights the potential of GNNs as structural indicators for motif mining.

Motif mining typically involves an additional significance testing step by comparing raw counts against randomized versions. In this work, we bypass explicit significance testing, investigating whether GNNs can directly learn representations to guide subgraph search. The remainder of this paper is organized as follows. Section 2 establishes the theoretical framework, detailing graphlet degree distributions, message-passing mechanics, and node-level feature representations, followed by our multilabel classification and homophily formalisms. Section 3 outlines the experimental setup, describing the dataset properties, label binarization scheme, and full hyperparameter search space. Section 4 presents the empirical findings across both transductive and inductive settings, examining the interplay between feature quality, aggregation operators, network depth, and oversmoothing, alongside the performance of our post hoc probability-guided motif search. Section 5 discusses the broader implications of embedding separability, topological limitations, and computational trade-offs and concludes this study with directions for future research.

## 2. Theoretical Framework and Modeling Approach

### 2.1. The Rudiments of Graph Theory

Motif finding inherently requires detecting whether target structures exist within a larger network via subgraph isomorphism. In this section, we first establish the foundational graph constructs that govern these matching tasks. A graph G=(V,E) models entities as vertices V(G) and their pairwise relationships as edges E(G)⊆V2, where two vertices u,v∈V are adjacent (u∼v) if {u,v}∈E [30]. At the local level, local message passing in GNNs operates over the open neighborhood N(v)={u∈V∣{u,v}∈E}, whose size determines the vertex degree deg(v)=|N(v)| [30]. To enable matrix-based GNN operations, *G* is compactly represented via its adjacency matrix $A\in R^{\left|V|\times |V\right|}$ [30,31], where

$$A\left(i,j\right)=\left\{\begin{array}{cc} 1, & \mathrm{if}\;v_{i}∼v_{j}\\ 0, & \mathrm{otherwise} \end{array}\right.$$

Building upon this matrix representation, motif discovery fundamentally relies on solving the NP-complete subgraph isomorphism problem [14,32] to identify whether a target motif *H* appears within *G*. Formally, H=(V(H),E(H)) is a subgraph of G=(V(G),E(G)) if V(H)⊆V(G) and E(H)⊆E(G). In the literature, motif mining is defined in subtly different ways depending on whether the target patterns are treated as general (non-induced) or induced subgraphs. Formally, given a subset of vertices W⊆V(G), the induced subgraph G[W] is defined as follows:

$$E\left(G\left[W\right]\right)=\left\{\{u,v\}\in E(G)|u\in W,v\in W\right\}.$$

To differentiate fine-grained structural positions within these induced motifs, vertex roles are classified via graph symmetries. An *automorphism* of a graph G=(V,E) is a self-isomorphism, defined as a bijection σ:V→V that preserves edge connectivity:

{u,v}∈E⇔{σ(u),σ(v)}∈E.

The set of all such bijections forms the automorphism group Aut(G). This group induces an equivalence relation over *V*, partitioning the vertex set into equivalence classes referred to as *orbits* [14,30].

In the remainder of this work, we use the terms vertex (plural: vertices) and node interchangeably.

### 2.2. Graphlet Degree Distribution

Formally, a *k*-graphlet denotes a small, connected, and non-isomorphic induced subgraph *g* comprising a subset of *k* interconnected nodes in *G* [14,33]. To account for structural symmetries within these subgraphs, automorphism orbits partition the nodes of a graphlet into equivalence classes, grouping together topologically equivalent positions that can be interchanged without modifying the overall graphlet structure [12,14,30]. As the subgraph size *k* grows, the combinatorial diversity of distinct undirected graphlets alongside their respective automorphism orbits grows exponentially. For instance, while a size of k=3 yields merely 2 unique graphlets and 3 orbits, increasing the order to k=5 expands this space to 21 graphlets with 58 distinct orbits, eventually scaling to more than two million orbits when considering graphlets of order k=9 [22,25].

The graphlet degree vector (GDV) of a node encodes how many times a node touches each graphlet at a particular automorphism orbit [12,14,22,25]. In this context, the node degree can be interpreted as a special case—namely, orbit o(0) [14]. For any given node, the total count for a specific graphlet is the sum of the orbit counts associated with that graphlet [12,22]. These vectors can be aggregated across the graph to form the graphlet degree distribution matrix ${\mathrm{GDD}}_{G}$, where entry (i,j) indicates the number of nodes that appear *i* times in orbit *j* [8,14]. The computational complexity of counting all graphlets of size *k* is $O(nd^{k-1})$, where *d* is the maximum degree of the graph *G*. Due to the combinatorial explosion, the computation for graphlets is usually restricted to k≤5 nodes [33,34,35]. Figure 1 shows graphlets of size up to five nodes.

### 2.3. Deep Learning on Graphs and Information Diffusion Mechanism

Graph neural networks (GNNs) leverage both the graph structure and node features $X_{v}$ to learn a representation vector for individual nodes $h_{v}$ or entire graphs $h_{G}$. Modern GNNs commonly follow a *neighborhood aggregation scheme*. After *k* iterations, the node embedding $h_{v}^{\left(k\right)}$ captures the structure of its *k*-hop neighborhood [21,36,37,38].

Formally, the general update rule at the *k*-th layer of a GNN is given by the following:

$$h_{u}^{\left(k+1\right)}={\mathrm{UPDATE}}^{\left(k\right)}\left(h_{u}^{\left(k\right)},{\mathrm{AGGREGATE}}^{\left(k\right)}\left(\{h_{v}^{\left(k\right)}|v\in N\left(u\right)\}\right)\right)$$

or equivalently,

$$h_{u}^{\left(k+1\right)}={\mathrm{UPDATE}}^{\left(k\right)}\left(h_{u}^{\left(k\right)},m_{N(u)}^{\left(k\right)}\right)$$

The AGGREGATE function collects information from the set of neighboring nodes N(u), resulting in a combined message $m_{N(u)}^{\left(k\right)}$ (Figure 2). Then, this message is passed to the UPDATE function, which combines it with the current embedding of node *u* to produce the new representation [21,28,36,37,38].

The initial embeddings at k=0 are set to the input features for each node:

$$h_{u}^{\left(0\right)}=x_{u}\;\forall u\in V$$

After running *K* iterations of the message-passing process, the final node embedding is obtained as follows:

$$z_{u}=h_{u}^{\left(K\right)}\;\forall u\in V$$

The neighborhood aggregation operation is fundamentally a set function. We are given a set of neighbor embeddings $\left\{h_{v}|\forall v\in N\left(u\right)\right\}$ and must map this set to a single vector $m_{N(u)}$.

A GNN must distinguish different multisets of node features—otherwise, it will map structurally different neighborhoods to the same embedding. Figure 3 illustrates this issue: although the graph structures around node *v* differ, simple aggregation schemes like *mean* or *max* assign the same embeddings to *v* and $v^{\prime }$. These functions fail to capture multiplicities in the multiset of neighbor features—they reduce the multiset either to its average or to its maximum element. This leads to an inability to distinguish graphs that differ only in neighborhood cardinality or composition. In general, the more discriminative the aggregation function is with respect to different multisets, the higher the representational power of the GNN [38].

### 2.4. Node-Level Features

Instead of computing embeddings for each node individually, GraphSAGE learns a function that can generate node embeddings by aggregating information from local neighborhoods [39]. This function can generalize to new nodes by leveraging node features. The method can also be applied to graphs without explicit node features, relying on structural signals instead. Node attributes are typically represented as a feature matrix $X\in R^{|V|\times m}$, where |V| is the number of nodes, and *m* is the dimensionality of the feature vectors. Basic statistics such as the degree of a node are frequently used as features in traditional node classification tasks. Further informative descriptions can be derived from the centrality measures, which quantify different aspects of a node’s importance within the graph structure [21,40].

Centrality measures estimate how important a node or edge is for maintaining connectivity or facilitating information flow [21,40]. The concept of centrality depends on the specific type of influence under investigation. Three widely used measures include:

- Degree centrality: Describes the number of direct connections a node has.
- Betweenness centrality: Describes how often a node lies on the shortest paths between other nodes.
- Closeness centrality: Defined as the inverse of the average shortest path from a node to all others.

The *eigenvector centrality* is defined recursively as follows:

$$e_{u}=\frac{1}{\lambda }\sum_{v\in V}A\left[u,v\right]\cdot e_{v}\;\forall u\in V$$

where λ is the leading eigenvalue of the adjacency matrix *A*, and e is the corresponding eigenvector representing node centralities [21,40].

Clustering captures the tendency of a node’s neighbors to also be connected to each other, forming triangles [20,21,41,42]. The local clustering coefficient $c_{i}$ for a node *i* with degree $k_{i}$ is defined as the ratio between the number $E_{i}$ of existing edges between the $k_{i}$ neighbors and the possible total number $\frac{k_{i}\left(k_{i}-1\right)}{2}$:

$$c_{i}=\frac{2\cdot E_{i}}{k_{i}\left(k_{i}-1\right)}$$

### 2.5. Cospectrality and Eigen Decomposition Pruning (EDP)

Two graphs are *cospectral* if they share the exact same adjacency eigenvalues. While all isomorphic graphs are inherently cospectral, the converse does not necessarily hold [30,43,44]. Spectral similarity is based on matrix similarity: two matrices $A_{1}$ and $A_{2}$ are similar if there exists an invertible matrix *P* such that the following holds:

$$A_{2}=P^{-1}A_{1}P.$$

If $A_{1}$ and $A_{2}$ are similar, then they share the same eigenvalues [30,31,43,44,45].

Verifying cospectrality does not require full eigenvalue decomposition. Instead, it is sufficient to demonstrate that the traces of powers of their adjacency matrices are equal across all walk lengths:

$$\mathrm{tr}\left(A_{1}^{k}\right)=\mathrm{tr}\left(A_{2}^{k}\right)\;\forall \;1\leq k\leq n.$$

Here, $\mathrm{tr}(A_{1}^{k})$ corresponds to the total number of closed walks of length *k* in the graph. Thus, $G_{1}$ and $G_{2}$ are cospectral if and only if for each k∈{1,2,…,n}, the total number of closed walks of length *k* in $G_{1}$ equals that in $G_{2}$ [30,44].

Building on spectral properties, Moayed et al. [46] introduced Eigen Decomposition Pruning (EDP) to pinpoint candidate subgraphs in large networks that potentially match a given query motif. The method is based on the following key concepts:

- Neighborhood Pattern p(u,k): Defined as the structural signature of the *k*-hop neighborhood around a node *u*.
- Neighborhood Subgraph ${\mathrm{NS}}_{G}^{k}\left(v\right)$: The induced subgraph containing all nodes and edges within a distance *k* from a node *v*.
- Local Laplacian Matrix (LLM):
  $$L_{L}\left({\mathrm{NS}}_{G}^{k}\left(v\right)\right)=D\left({\mathrm{NS}}_{G}^{k}\left(v\right)\right)-A\left({\mathrm{NS}}_{G}^{k}\left(v\right)\right),$$
  where *D* and *A* denote the degree and adjacency matrices of the induced subgraph, respectively.

Inspired by the EDP method, we approximate the Laplacian spectrum of each node’s 2-hop egonetwork using the *ApproxSpectralMoment* algorithm proposed by [45,47]. This method estimates the eigenvalue distribution by simulating 10.000 random walks of length 20 and computing the proportion of closed walks, which reflect the spectral properties of the transition matrix. For each egonetwork, we repeat the estimation 20 times and average the results. From the resulting spectra, we extract the first six spectral moments per node. Since the first moment is zero, we use moments 2–6 as features. These moments are isomorphism-invariant, meaning they remain unchanged for structurally equivalent graphs [45]. We use the intersection of the set of spectral features of the query graph and subgraphs as a clue of similarity.

This spectral approach is further validated by Jin and Zafarani [48], who established formal equivalences between low-order spectral moments and fundamental node attributes (e.g., degree and clustering coefficient) as well as motif patterns such as edges, wedges, triangles, and cycles.

### 2.6. Multilabel Classification

*Multilabel classification* is a generalization of binary classification to multiple independent labels [49]. Each instance is associated with a subset of labels S⊆L, so the dataset D becomes the following:

$$D=\{\left(x_{1},S_{1}\right),\left(x_{2},S_{2}\right),\ldots ,\left(x_{n},S_{n}\right)\}$$

The binary relevance method trains |L| binary classifiers $C_{1},C_{2},\ldots ,C_{\left|L\right|}$, where each classifier is responsible for predicting the presence or absence of its corresponding label [49,50]. In the multilabel setting, the following F1 variants are commonly applied: micro, macro and instance-based averaging [49,51]. In practice, each label can be assigned a separate decision threshold. Here, we assume a fixed probability cutoff (e.g., 0.5) to make the approach more reproducible.

### 2.7. Graph Homophily

Label homophily quantifies the similarity among neighboring nodes based on their label distributions. Graphs are typically categorized as either homophilic or heterophilic [36,50,52].

Node homophily $H_{node}$ is defined as the average proportion of the edge label consistency of all the nodes [36,50,52]. Formally, we have the following:

(1) $$H_{node}=\frac{1}{\left|V\right|}\sum_{v\in V}\frac{\left|\{u\in N\left(v\right):y_{v}=y_{u}\}\right|}{\left|N(v)\right|},$$

where V is the node set, and N(v) is the neighbor set of node *v*. $y_{v}$ and $y_{u}$ denote the labels of nodes *v* and *u*, respectively. Gong et al. [36] extend this definition to k-hop neighbors to measure high-order homophily:

(2) $$H_{high-order}=\frac{\left|\{u\in N^{\left(k\right)}\left(v\right):y_{v}=y_{u}\}\right|}{|N^{\left(k\right)}\left(v\right)|}$$

where $N^{\left(k\right)}\left(v\right)=\left\{u:1\leq \mathrm{ShortestPath}\left(u,v\right)\leq k\right\}$ represents the k-hop neighbors of *v*. Both node and edge homophily are in the range of [0, 1], and high homophily indicates low heterophily and vice versa [36].

Zhao et al. [50] argue that the basic homophily definition cannot be directly used in multilabel graph datasets, as each node can have more than one label. Further, they define multilabel label homophily as follows:

Given a multilabel graph G, the label homophily h of G is defined as the average of the Jaccard similarity of the label set of all connected nodes in the graph:

(3) $$h=\frac{1}{\left|E\right|}\sum_{(i,j)\in E}\frac{\left|l(i)\cap l(j)\right|}{\left|l(i)\cup l(j)\right|}.$$

### 2.8. Oversmoothing in Graph Neural Networks

When striving for generalization, it is important to account for oversmoothing—a phenomenon where deep GNNs tend to produce almost constant representation, independent of the original node feature distribution, which can ultimately lead to the loss of discriminative power [53,54,55,56,57,58,59]. Formally, let $H^{\left(\ell \right)}\in R^{n\times d}$ be the matrix of node embeddings after *ℓ* message-passing layers in a GNN, where *n* is the number of nodes, and *d* is the hidden dimension. Consider a similarity function π:H→R, where $H=R^{n\times d}$ is the space of all possible embedding matrices. We say that a deep GNN experiences oversmoothing if

$$\lim_{\ell \to \infty }\pi \left(H^{\left(\ell \right)}\right)=c,$$

where *c* is a constant indicating the collapse of embeddings [52,54]. This creates an almost constant representation, independent of the original node feature distribution [53,54,55,56,57,58,59].

In the literature, a drop in test accuracy in deep GNNs is frequently blamed on oversmoothing, following the hypothesis that once embeddings collapse into a trivial space, nodes become hard to separate, leading to a decline in performance [54,55]. To pin this down, recent studies have worked on more precise definitions of oversmoothing using metrics like graph Dirichlet, cosine similarity, or mean-average distance (MAD) [54,59]. To determine how far information needs to propagate, Alon and Yahav [53] define a “problem radius” *r*, which is usually approximated by tuning the number of layers *K*. In practice, since most nodes in a connected component are within a few hops of each other, GNNs with just two or three layers are often sufficient to achieve competitive results [53,58].

While Chen et al. [56] attribute oversmoothing to the overmixing of signals—where inter-class interactions introduce harmful noise and impair representation separability—the nature of our task introduces a unique constraint. Because our target labels are inherently tied to the network topology, high local label density is practically unavoidable. Consequently, we hypothesize that this task requires only a short receptive field, as the underlying graphlet structures span a maximum neighborhood of two hops. Nonetheless, to examine how GNN performance scales with network depth, we evaluate architectures ranging from 2 to 12 layers—extending one layer beyond the diameter of the interactome. We systematically investigate how label homophily varies across different graphlets as network depth increases and how these structural changes impact performance. In the literature, various architectural techniques—such as residual connections, normalization layers, or gating mechanisms—are commonly used to mitigate oversmoothing and preserve local distinctions [54,57,59]. Building on this, we specifically incorporate batch normalization and dropout into our models to stabilize training and counteract embedding collapse across these varying depths.

## 3. Experimental Setup

### 3.1. Dataset Description

In this study, we used the human proteome-scale PPI network published by Thul et al. [7], accessible via the Human Cell Map. The network was modeled as an undirected graph, and for all subsequent analyses, we extracted its greatest connected component (GCC) containing 7185 nodes and 59,968 edges. For training and validation in the inductive setting, we employed the BioSNAP human PPI dataset [60], which contains 24 biologically meaningful networks.

### 3.2. Label Distribution

ORCA provides a 73-component graphlet GDV for each node in the graph. To establish our ground truth, we convert the GDV into a count vector and binarize it to indicate simply whether a node participates in a given graphlet or not. To quantify class prevalence, we define the normalized label frequency by dividing the count of active nodes by the total number of nodes |V| for each of the 29 labels. Figure 4 illustrates the normalized label frequency for graphlets 1 through 29 (orbit 0 and graphlet 0, corresponding to a simple edge, are omitted). The normalized label frequency exhibits pronounced class imbalance, an inherent consequence of explicitly searching for induced subgraphs.

### 3.3. Model Architecture and Hyperparameter Space

Each GNN layer in this framework can be defined as follows:

$$h_{v}^{\left(k+1\right)}=\mathrm{AGG}\left(\left\{\mathrm{ACT}\left(\mathrm{DROPOUT}\left(\mathrm{BN}\left(W^{\left(k\right)}h_{u}^{\left(k\right)}+b^{\left(k\right)}\right)\right)\right):u\in N\left(v\right)\right\}\right)$$

where $h_{v}^{\left(k\right)}$ is the *k*-th layer embedding of node *v*, $W^{\left(k\right)}$ and $b^{\left(k\right)}$ are trainable weights, and N(v) is the local neighborhood of *v*. We stack multiple layers and define the full model configuration as the Cartesian product of the following intra-layer design options:

- Aggregation function: {mean,max,sum}.
- Activation function: {ReLU}.
- Batch normalization: {True,False}.
- Dropout rate: {0.0,0.1,0.2}.
- Hidden dimension: {128,256}.

We evaluate architectures ranging from 2 to 12 layers. This results in a total of 11×3×1×2×3×2=396 unique configurations, each of which was trained for 1000 epochs per feature set. To ensure robustness and account for initialization variance, every configuration was evaluated using five different random seeds. All models were trained using the Adam optimizer with a fixed learning rate of 0.001, complemented by early stopping and weight decay. We used GraphSAGE as the base model for its inductive capabilities [29,39]. The simulation environment was implemented in Python (Python 3.10.16), using widely adopted graph learning libraries such as PyTorch Geometric (version 2.6.1) and NetworkX (version 3.4.2), together with standard packages like NumPy (2.2.5), Matplotlib (3.10.9), and Pandas (2.2.3). For the multilabel classification task, we used the BCEWithLogitsLoss function, which combines a Sigmoid layer and the binary cross-entropy loss in a single class. This enables the model to make independent binary predictions for each graphlet membership per node.

## 4. Results

### 4.1. Results in Transductive Setting

#### 4.1.1. Grid Search and Effect of Varying Feature Quality

To analyze the mechanics of model depth and feature representations, we conducted a full grid search across 396 architectural configurations over five random seeds. As illustrated in Figure 5, the decline in performance with increasing layer depth is strongly dependent on the choice of the aggregation operator and the utilized feature set. Specifically, our results demonstrate that the max, mean, and sum strategies react differently to increased depth, suggesting that the observed performance degradation is not merely a consequence of the number of layers (i.e., ”excessive smoothing”) but rather a result of the specific mechanism of information aggregation and its interaction with the input features.

Figure 6 reports the mean macro F1 performance across all feature sets, where error bars denote the standard deviation across configurations sharing the same design choices. In alignment with Figure 5, performance fluctuates heavily with network depth. This variance is reflected in the large standard deviations observed in Figure 6, particularly for deeper architectures where representation collapse occurs. As the network depth increases from 2 to 12 message-passing layers, models consistently suffer from degradation due to overfitting and embedding collapse. However, the severity of this performance drop varies significantly depending on the choice of aggregation operator and feature informativeness:

- **Sum Aggregation and Expressiveness Trade-offs:** While sum aggregation enhances expressiveness for uninformative or dummy features (e.g., constant scalar) by preserving multiset cardinalities, it becomes inherently unstable with increasing depth. Because the receptive field grows exponentially with node degree, summing unnormalized neighbor features leads to an uncontrolled accumulation of ones, causing training loss to surge drastically and remain stuck at high levels.
- **Hidden Dimensions and Feature Similarity:** Model performance remains virtually on par across different hidden dimensions (128 vs. 256).
- **Dropout Impact:** By randomly zeroing out features during message passing, dropout disturbs consistent neighborhood aggregation. Under sum aggregation, this introduces violent amplitude fluctuations, whereas under max aggregation, it severely exacerbates information loss.
- **Batch Normalization:** The impact of batch normalization fluctuates strongly based on the aggregation function. While it acts as a crucial stabilizer that prevents gradient explosion under sum aggregation and mitigates oversmoothing, its interaction with max and mean aggregations shows varying dynamics across hyperparameter choices (see Appendix B for details).

To analyze the underlying mechanics of oversmoothing, we examine how feature distributions evolve across network layers. Formally, a GNN reaches a state of complete representation collapse ($\mu (X^{\left(l\right)})=0$) when its covariance matrix contracts to constant entries, $K_{\alpha \beta }^{\left(l\right)}=k^{\left(l\right)}$ [57]. Under this condition, nodes receive identical pre-activations, ultimately collapsing into uniform embeddings ($x_{\alpha }^{\left(l\right)}=x_{\beta }^{\left(l\right)}$). As demonstrated in prior work, this uniform state acts as an invariant fixed point of the network dynamics [57]. Driven by strong layer-wise contraction properties, this fixed point behaves as a powerful attractor: even when starting from distinct initial features ($d(x_{\alpha }^{\left(0\right)},x_{\beta }^{\left(0\right)})\neq 0$), node representations rapidly converge toward zero feature distance ($\mu (X^{\left(l\right)})\to 0$) as network depth increases [57].

To empirically evaluate this behavior, we recorded the final node embeddings across all evaluated network depths (2→12) and computed their pairwise cosine similarities (Figure 7). While several configurations exhibit a steep increase in similarity toward higher depths—indicating severe embedding collapse—our findings show that this effect is highly sensitive to model architecture. In particular, **batch normalization (Batch Norm)** acts as a vital stabilizer across all aggregation schemes: by re-scaling and re-centering features at each step, it continuously reintroduces variance into the feature space, thereby suppressing oversmoothing and maintaining layer-wise feature separability.

Building upon this theoretical framework, our grid search reveals an intriguing interplay between feature informativeness and aggregation methods:

- **Constant Scalar:** Homogeneous initializations, such as a constant value of 1 assigned to every node, places the network almost directly onto the fixed point, maximally accelerating the collapse of representations.
- **Spectral Moments and Centralities:** Spectral moments derived from 2-hop egonetworks exhibit highly overlapping neighborhoods due to the interactome’s high clustering coefficient.
- **Centralities and Clustering Coefficient:** Centrality measures paired with the clustering coefficient provide node-specific attributes, offering the highest degree of distinguishability and performing the best.
- **Aggregation Function:** max-pooling proves to be the weakest and most unstable aggregation function, consistently characterized by aggressive information loss and the rapid collapse of representations. sum aggregation acts as a strong contraction driving the network toward the attractive fixed point, though it can theoretically prevent collapse when paired with proper weight variance tuning ($\sigma_{w}^{2}$) [57]. In contrast, mean aggregation excels when paired with informative input features, acting as a stabilizing damper through constant degree normalization.

Finally, introducing **dropout** modulates this behavior significantly depending on the chosen aggregation scheme. Dropout acts as a regularizer but, in deep GNNs, leads to stochastic feature dropping that accelerates embedding collapse:

- Under sum aggregation, which scales neighbor messages directly by the node degree, dropout breaks the consistent accumulation of signals, resulting in violent amplitude fluctuations and a rapid drift into the attractive fixed point.
- max aggregation inherently preserves only the dominant neighborhood signal while discarding all alternative features. Consequently, random feature dropping severely compounds this information bottleneck, depriving the network of sufficient variance and causing rapid representation degeneration.
- Conversely, mean aggregation remains robust against dropout disturbances. By dividing by the node degree, it compensates for missing signals and prevents the abrupt breakdown of the information flow, as further evidenced in Figure 7.

For a detailed breakdown of results across varying dropout rates and hidden dimensions, we refer to Appendix B.

Figure 8 reports the graphlet precisions averaged over five train/val/test splits, highlighting clear performance variations across both feature sets and aggregation mechanisms. The anticipated failure cases are prominently reflected in the constant scalar setting, where cycles (e.g., $G_{2},G_{5},G_{15}$) and clique graphs ($G_{8},G_{29}$) exhibit vanishing precision. Theoretically, these non-isomorphic topologies cannot be distinguished through local feature distributions alone (Figure 3), as they differ strictly in neighborhood cardinality and composition.

Under uninformative inputs, less expressive, non-injective aggregators (e.g., mean or max pooling) map these distinct local neighborhoods onto identical vectors, inducing severe embedding collapse. In contrast, sum aggregation preserves multiset injectivity by accumulating unscaled neighbor signals, thereby retaining neighborhood cardinality and enabling the model to disambiguate subtle structural differences. Beyond these expressiveness limitations, a comparison with the label distribution (Figure 4) reveals that class imbalance further amplifies underperformance, as graphlets with sharply declining precision values are heavily underrepresented in the interactome.

#### 4.1.2. Effect of Label Homophily

A complementary explanation for the precision fluctuations in Figure 8 is oversmoothing, caused by information overmixing across layers. To analyze this, we extend our evaluation to higher-order homophily based on Gong et al. [36], adapting their formulation to our multilabel setting by computing the metric independently for each label across *k*-hop neighborhoods (k∈{1,…,11}). As shown in Figure 9, this reveals distinct topological patterns: graphlets like $G_{2}$, $G_{5}$, and $G_{15}$ show low local homophily (k=1) that decays rapidly over higher hops. In these regions, nodes aggregate signals from neighbors with different labels, blurring feature boundaries. For graphlet classification, this is worsened by non-induced neighbor connections: nodes within an induced graphlet often connect to non-induced variants of the same motif, pulling in signals from “forbidden” structures. Combined with their low representation in the dataset (Figure 4), this lack of neighborhood consistency and structural noise prevents the model from separating classes effectively, causing sharp precision drops for minority graphlets in deeper networks.

### 4.2. Results in Inductive Setting

#### 4.2.1. Grid Search and Effect of Varying Feature Quality

To evaluate the model’s ability to generalize to unseen graph structures, we adapted the experimental design for the inductive setting by constraining the network depth to 2–4 layers. Table 1 shows the condensed configuration space for the inductive setting. The dataset comprises a heterogeneous collection of BioSNAP and cell compartment graphs, varying significantly in size and density (see Table A1), as well as label frequency (see Figure A1).

To ensure balanced representation across network scales, we partition the sorted graphs into three size bins (11 small, 11 middle, 11 big). Across five seeds, each bin is independently shuffled to prevent structural bias. For every seed, the dataset is stratified into train, validation, and test splits by drawing fixed proportions from each size tier:

- **Train:** Comprising 6 small, 6 middle, and 5 big (17 total).
- **Val:** Comprising 3 small, 3 middle, and 2 big (8 total).
- **Test:** Comprising remaining samples from each tier (8 total).

This binning strategy seeks to maintain proportional coverage across all graph scales, reducing the risk of size-induced distribution shifts during inductive evaluation.

Figure 10 presents the hyperparameter landscape across the configuration space. Compared to the transductive setting, the performance profiles appear markedly more stable, as the constraints on network depth effectively prevent the inclusion of severely oversmoothed configurations.

While the qualitative preferences—such as the inherent instability of max aggregation and the sensitivity to feature informativeness—persist, the inductive performance highlights two key observations:

- **Optimal Depth:** All three feature sets maintain their strong preference for shallow, two-layer architectures, confirming that depth optimization is an intrinsic property of the feature set rather than a setting-specific artifact.
- **Aggregation Dynamics:** While sum and mean aggregations perform nearly on par when utilizing informative Spectral Moments and Centralities, the sum operator consistently outperforms mean for Constant Scalar inputs.

While these results corroborate the findings from our transductive analysis, the inductive setting introduces distinct peculiarities, particularly regarding topology-dependent normalization instability. Figure 11 illustrates the impact of inductive BatchNorm (orange) compared to models without normalization (blue) across the feature sets. The x-axis displays aggregated BioSNAP test graphs alongside four specific cell organelle test graphs (Cytoplasm, Nucleus, Golgi, Vesicles), measured by the mean macro F1 score. While the BioSNAP benchmarks exhibit very high and stable median performance, activating BatchNorm reveals a prominent distribution of deep outliers dropping to values of 0.45.

As outlined by Cai et al. [61], inductive BatchNorm computes statistics dynamically per mini-batch. When individual samples inadequately represent the heterogeneous diversity of graph topologies in the entire dataset, the mismatch between training and inference statistics results in feature distortion and unstable optimization. Since the dataset is dominated by large, homophilic graphs alongside only a few small, topologically distinct graphs with lower homophily, normalization across features leads to substantial “batch noise” [61]. While performance on structural outliers (such as Golgi) remains consistently constrained across both variants, the primary vulnerability introduced by BatchNorm manifests in the BioSNAP benchmarks: here, normalization triggers severe optimization instability and a prominent long tail of deep outliers that are absent in the unnormalized models. In contrast to the transductive setting—which relies on global neighborhood contexts and consistent statistics—the inductive setting suffers from unchecked instance variance whenever the graph topology deviates sharply from the expected distribution [61]. Because we utilize features derived from the topology, this effect becomes particularly pronounced: Even with uninformative features, neighborhood aggregation correlates strongly with the node degree. When batch normalization normalizes across features here, these structural details are distorted, significantly amplifying the observed instability and deep outliers.

The label-wise precision plot in Figure 12 reveals a marked reduction in the performance drops previously observed in the transductive setting. Benefiting from a substantially larger and more diverse pool of training data points, the GNN achieves smoother and more consistent precision profiles across label classes. Nevertheless, the Constant Scalar features continue to exhibit characteristic performance drops across specific failure cases, heavily dependent on the chosen aggregation method. A potential explanation for the observed weaknesses of Spectral Moments comes from the local nature of their computation. Because Spectral Moments are derived from bounded subgraphs (e.g., 2-hop egonetworks), adjacent nodes share a substantial portion of their local neighborhood. Consequently, neighboring nodes receive highly correlated initial feature representations. This spatial overlap renders the representations particularly vulnerable to accelerated oversmoothing during message passing.

#### 4.2.2. Effect of Label Homophily

Table 2 outlines the structural characteristics and the macro F1 performances of the best configuration across the test set, with overall performance closely tracking the general mean trend, highlighting three core insights into inductive generalization:

- **Stable Generalization under High Homophily:** The BioSNAP graphs exhibit exceptionally high label homophily (≈0.96–0.98) alongside very low Jensen–Shannon divergences (JSDs). Under such extreme homophily, message passing acts primarily as a smoothing operator over aligned labels, mitigating misclassifications from oversmoothing. The minimal Jensen–Shannon divergence confirms that local neighborhood features remain topologically consistent. This structural consistency explains why macro F1 scores remain constantly high (≈0.97).
- **Impact of Graph Scale and Distribution:** While larger BioSNAP networks display near-perfect homophily and homogeneous label distributions, smaller or more structurally diverse graphs—such as *Golgi* (|V|=192) and *Vesicles* (|V|=585)—exhibit a noticeable drop in homophily and JSD values. This structural divergence correlates directly with lower macro F1 scores, particularly when paired with uninformative Constant Scalar features.
- **Robustness of Informative Features:** Even in structurally challenging environments, such as the *Golgi* compartment with a homophily of 0.81, Spectral Moments and raw node features maintain remarkable stability. They significantly outperform the Constant Scalar baseline, underscoring that informative feature engineering effectively mitigates performance losses induced by low homophily.

### 4.3. Graphlet Matching Guided by Predicted Probabilities

To complement the GNN predictions, we implement a post hoc motif detection strategy that explicitly counts motif instances. While classification models indicate whether a node participates in a specific motif, they do not quantify the exact frequency or local context of its occurrence. To address this limitation, we developed custom detectors for each of the 29 graphlets. Obtaining a reliable ground-truth count requires aggregating node-specific information; to this end, we leverage ORCA, which provides the frequency of a node *v* appearing in a given orbit o(x). Through combinatorial transformations, these orbit counts are converted into graph-wide totals: one can either sum the asymmetric orbit counts across all nodes or utilize symmetric orbit counts adjusted by the number of unique symmetric orbits within the respective graphlet.

The transition to k=5 graphlets introduces an algorithmic overhead, as it requires expanding the local search space to 2-hop neighborhoods and repeatedly evaluating structural constraints (“deal-breaker edges”) across larger neighborhoods. Unlike for k∈{3,4} graphlets—where typically 2 neighbors are selected simultaneously (with exceptions for $G_{4}$, $G_{6}$, and $G_{8}$)—the detectors for k=5 are designed to pick 3–4 neighbors at a time. This structural shift triggers a severe combinatorial explosion within the nested evaluation loops across larger graphs. By applying a trained GNN to the test graphs, our hybrid framework introduces a flexible time–precision trade-off. However, because the BioSNAP graphs exhibit high label frequency, the probability-guided detectors target nearly every node for the search, making it functionally equivalent to an exhaustive search in practice. Consequently, we intentionally restricted our quantitative analysis to k∈{3,4} graphlets.

Detection is simulated for each feature set using its best-performing configuration {Layers, Aggregation., Batch Norm., Dropout, Hidden Dim.} based on the macro F1 score:

- **Constant Scalar:** {2,sum,False,0.0,128}.
- **Spectral Moments:** {2,sum,False,0.1,256}.
- **Centralities and Clustering Coefficient:** {4,mean,True,0.1,256}.

For each graphlet, target nodes exceeding a classification probability threshold of 0.5 are assigned an anchor role (orbit) based on their local neighborhood. We then extract their 1-hop neighborhood and filter out candidate nodes that violate structural constraints by containing “deal-breaker” edges—connections explicitly prohibited within the target graphlet’s induced subgraph definition (see Figure A5). For a detailed overview of the absolute errors across feature sets and test graphs, see Table A3, Table A4 and Table A5 in Appendix C.

Table 3 presents the ground-truth counts computed via ORCA alongside the absolute deviation from our detected counts. Consistent with Figure 8, the absolute differences indicate that graphlets with lower precision were omitted more often; however, these deviations are several orders of magnitude below the true counts.

To limit computational complexity, our workflow replaces exhaustive search with a targeted orbit-matching strategy, identifying positive nodes only when positioned on detector-suited orbits. Integrating an analytical counting algorithm, a GNN, and heuristic detectors, we search for graphlets across 21 test graphs. Missed graphlet instances primarily stem from misclassified orbits that serve as anchor nodes for the detector (see Figure A4).

Although we initially intended to use GNN-predicted probabilities to bypass exhaustive mining, high local homophily in PPIs causes probability-guided search to closely mimic exhaustive search in practice. Nevertheless, because our detector selectively targets nodes lacking prohibited “deal-breaker” edges, subtle runtime gains may still exist. As demonstrated by Hočevar and Demšar [25], ORCA scales exceptionally well on sparse graphs. However, its exact counting mechanism relies on a polynomial dependency on the maximum node degree ($O(nd^{3})$), making it highly sensitive to dense regions and combinatorial explosions when encountering networks with massive super-hubs. In contrast, a trained GraphSAGE model circumvents local density bottlenecks entirely through neighborhood sampling. Its per-batch space and time complexity is fixed at $O\left(\prod_{i=1}^{K}S_{i}\right)$ across *K* layers (where $S_{i}$ are user-specified sample sizes), completely decoupling the inference runtime from the true local node degree *d* [29]. This architectural advantage comes with a critical limitation: GraphSAGE achieves its superior scalability at the cost of an approximation trade-off.

A formal time complexity comparison incorporating explicit sampling strategies remains beyond the scope of this work. Furthermore, formal statistical significance testing to isolate true motifs is not viable under this setup, as the remaining predictions preserve inherent uncertainty. These directions are left for future research.

## 5. Conclusions

In this study, we investigated the capabilities and limitations of GNNs in solving subgraph-related tasks. We demonstrated that while GNNs exhibit strong performance in common tasks such as node classification or link prediction, their expressiveness falls short when it comes to more complex structural reasoning tasks, such as subgraph isomorphism or graphlet counting. Ultimately, learning such induced substructures is governed by a multifaceted interplay rather than a single bottleneck. By leveraging foundational metrics like homophily and initial embedding separability, we map out these performance variations through the following key takeaways.

**Oversmoothing and Feature Separability.** Oversmoothing remains a heavily debated topic in the GNN literature. While studies largely agree that oversmoothing is a widespread phenomenon, the challenge of oversmoothing and overmixing in GNNs depends heavily on evaluation metrics and architectural variants (such as GCN, GAT, GIN, and GraphSAGE), as demonstrated by Arnaiz-Rodríguez and Errica [54]. However, low accuracy in deep GNNs cannot be attributed to representation collapse alone. Instead, initial node embedding separability plays a decisive role alongside training pathologies such as vanishing gradients or overfitting [54]. Remedies like skip connections, normalization, or gating are standard architectural tools designed to preserve local distinctions [54,57,59]. Beyond standard architectural tools, alternative strategies focus on actively restructuring the learning environment. For instance, Chen et al. [56] mitigate overmixing by optimizing graph topology—removing inter-class edges and adding intra-class edges based on gold labels. Conversely, Rusch et al. [59] conclude that a major pitfall in designing deep GNNs that combat oversmoothing is sacrificing expressive power merely to keep node similarity measures approximately constant. In our experiments, oversmoothing emerges as an intricate interplay between feature informativeness, network depth, and the configuration space. Notably, higher initial feature similarity drastically accelerates the onset of embedding collapse, shifting optimal performance toward shallower architectures.

**Interplay of Depth, Topology, and Homophily.** We evaluate a non-trivial task where feature distributions across classes partially or completely overlap, placing structural topology at the center of the learning objective. In a short-range task characterized by high label homophily, we target induced structures within local neighborhoods, yielding correlated label distributions. By extending maximum network depth beyond the network diameter, we explicitly analyze performance decay under deep aggregation. Due to the small-radius nature of graphlet detection, shallow networks (2–3 layers) consistently achieve peak performance. However, classifying strictly induced structures introduces unique topological constraints: mutual exclusivity does not protect the model from information overmixing as depth increases. While classes with high local homophily build robust representations, low-homophily graphlets suffer from severe overmixing across inter-class and non-induced edges. As a result, the model aggregates signals from “forbidden” neighbor contexts, causing rapid oversmoothing and degraded precision for minority classes in deeper layers.

**Impact of Feature Quality and Network Models.** Biological graphs are real-world networks, alongside other domains such as social networks, the World Wide Web, or infrastructure networks. A defining characteristic of these networks is the scale-free property, which requires the careful handling of high-degree nodes (“hub nodes”) [62]. Utilizing domain-agnostic or dummy feature sets (e.g., a constant scalar) can enhance model generalizability, but it tightly couples performance to graph topology. In our inductive evaluations, this topological dependency became particularly evident: smaller, more densely clustered networks with higher Jensen–Shannon divergence consistently yielded significantly weaker results than medium and large graphs. Remarkably, in these medium-to-large networks, the constant scalar feature performed almost on par with more expressive feature sets, as neighborhood summation effectively allows the model to recover structural proxies like node degree. However, relying purely on constant features limits inductive transferability and leaves scalability questionable, as unnormalized summation over expanding receptive fields introduces size and magnitude effects in larger graphs. Historically, numerous graph architectures have been proposed to emulate the topological properties of real-world networks. However, standard synthetic network models—such as Erdős–Rényi or Watts–Strogatz graphs—exhibit fundamentally different degree distributions, clustering behaviors, and graphlet counts compared to biological networks [20,41,62]. Consequently, topology-dependent features may struggle to generalize across distinct graph families.

**Aggregation and Regularization Mechanics.** We propose a full-batch approach operating without neighborhood sampling that maintains strong discriminative capacity even under dummy feature regimes. While GraphSAGE’s sampling strategy could potentially mitigate size-related effects and enhance generalizability in an inductive setting, feature informativeness introduces a non-negligible time–performance trade-off. A crucial interplay emerges between network topology, feature expressiveness, and the choice of aggregation function (mean, max, and sum). Because our models operate on very small feature sets, traditional regularization techniques exhibit counterproductive dynamics. Specifically, applying dropout on low-dimensional input vectors disturbs neighborhood aggregation consistency and severely accelerates oversmoothing. Conversely, batch normalization acts as a vital stabilizer: by mitigating feature alignment and managing amplitude explosions under sum aggregation, it counteracts the initial high feature similarity and preserves embedding separability across layers. As Cai et al. [61] demonstrate, graph datasets suffer from significant “batch noise” compared to other domains, because single samples rarely capture the diverse topological structures of the whole dataset. Building on this, we observe that in our inductive setting—which is dominated by large, homophilic graphs—BatchNorm fails to generalize effectively, becoming highly unstable across features. While normalization variants like GraphNorm seek to alleviate such distribution instabilities, alternative optimization and refinement strategies are required to prevent graph aggregation from propagating flawed or diluting signals. Although attention mechanisms are increasingly tailored to address homophily bottlenecks—such as vanilla GATs, recent refinements like Signed Attention (SAGNN) [63], and supervised homophily losses [64]—they remain bound by permutation invariance and the 1-WL expressive limit. Consequently, rather than introducing heavy hyperparameter overhead and vulnerability in inductive settings, we rely on simpler aggregation methods that yield more stable and interpretable trajectories for capturing structural motifs.

**Substructure Localization and Post Hoc Search.** While GNNs excel at similarity-based tasks like node classification, they struggle with explicit substructure localization and counting. Existing methods typically reduce subgraph matching to binary node classification, labeling query nodes as +1 and remaining nodes as −1, which localizes substructures but fails to resolve exact node-to-node correspondences [65]. We address this limitation using post hoc, probability-guided detectors. A key advantage of our proposed pipeline is its dynamic design. PPIs are inherently dynamic, continually evolving—either through newly discovered interactions or by merging sources (e.g., HPRD, BioGRID, STRING) to complete the interactome. At the same time, handling PPI data requires caution: techniques like yeast two-hybrid screening carry high false-positive rates. To ensure reliable downstream hypothesis testing, researchers must often filter out these spurious edges, further altering the graph topology [10]. To handle these topological changes, we selected GraphSAGE, an inductive GNN framework. Dynamic node additions from database integration or removals from quality filtering are easily accommodated, as the model generates new predictions on the updated graph without requiring retraining. To avoid an exhaustive search across the entire network, predictions can be restricted to a local egonetwork around the affected nodes. However, high label homophily in real-world interactomes causes probability-guided searches to closely mimic exhaustive searches in practice, offering limited relief at high label densities. Although our hybrid detector does not yield a general-purpose complexity breakthrough for arbitrary graph types, it successfully transforms brute-force motif mining into a targeted refinement process with high precision in inductive settings. Future work should evaluate synthetic benchmarks with controlled label densities to formally delineate the time complexity boundary between probability-guided and exhaustive search strategies.

**Expressivity Trade-offs and Open Challenges.** Importantly, we showed that current GNN architectures still rely heavily on heuristic supervision or ground-truth annotations for tasks like subgraph matching. No automatic matching strategy has yet emerged that can reliably recover isomorphic structures in a general setting. While our ground-truth generator, ORCA, provides exact instance counts for a given node *v*, it shares a fundamental reliance on a subsequent search mechanism—with the crucial distinction that ORCA provides a priori knowledge of node *v*’s exact graphlet or orbit participation beforehand. The counting complexity for computing all k=4 orbits scales as $O(nd^{3})$ (where *n* is the number of nodes, and *d* is the maximum degree), meaning computational cost grows steeply with network size [25]. By applying a trained GNN to test graphs, our hybrid framework introduces a flexible time–precision trade-off. Our inclusion of spectral, centrality-based, and constant scalar features provided a thorough feature-based benchmarking, underscoring the trade-offs between model depth, expressivity, and task-specific performance. Our experiments revealed that even with minimal or noisy input features, GNNs are capable of learning useful representations. However, challenges such as oversmoothing, limited receptive field adaptation, and the inability to produce fine-grained node-to-node mappings persist. Structural variability across distinct topological profiles amplifies the time–precision trade-off, leading to the core insight that achieving meaningful time complexity relief in hybrid graphlet matching fundamentally relies on a dual mechanism: targeting networks with sparser label homogeneity to accelerate counting and matching while simultaneously ensuring structural conditions that preserve the GNN’s generalization capacity. As demonstrated in our inductive evaluation, topological variability introduces significant challenges for model generalization, showing that GNN optimization is a highly non-trivial task that directly dictates downstream precision. Depending on this attained precision and its interplay with label homogeneity, the hybrid technique dynamically fluctuates between an exhaustive search and targeted subgraph sampling. Designing or generating bespoke, topology-tailored graph benchmarks to systematically optimize this dual objective represents a comprehensive study in its own, which we leave for future research.

While the proposed experiment functions as a dry-run for employing geometric deep learning on complex tasks like subgraph matching, we find that topology is a persistent undercurrent in GNN training. Even as state-of-the-art methods strive to optimize feature separability, homophily, and message aggregation, topology proves to be a vital stumbling block that requires careful management to ensure high generalizability. In conclusion, while GNNs remain a powerful tool in graph representation learning, their application to motif-centric tasks requires further architectural and theoretical advances.

## Figures and Tables

**Figure 1:**
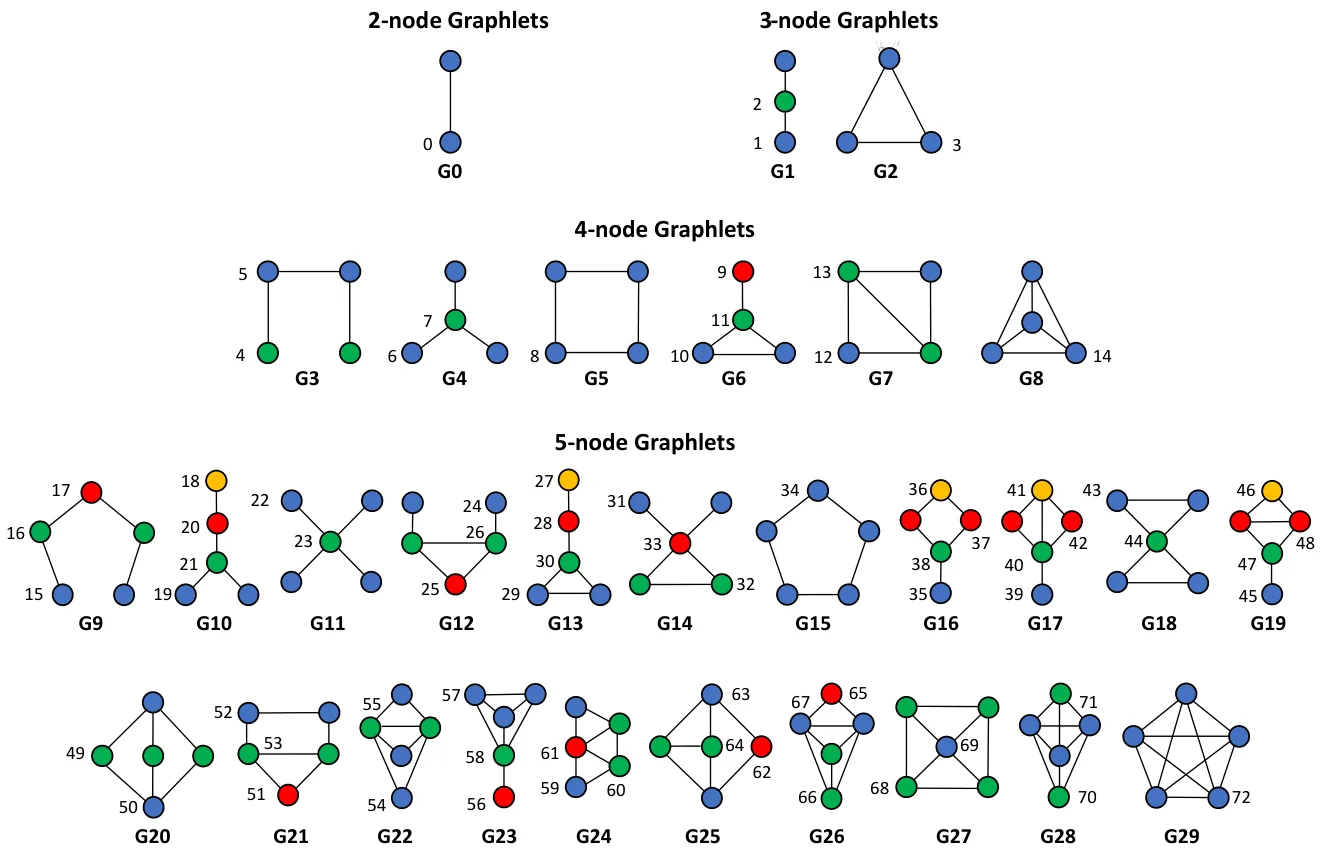
Non-isomorphic connected graphlets of size up to five nodes. Different colors represent distinct automorphism orbits, reflecting structural node roles. (Source: adapted from [25].)

**Figure 2:**
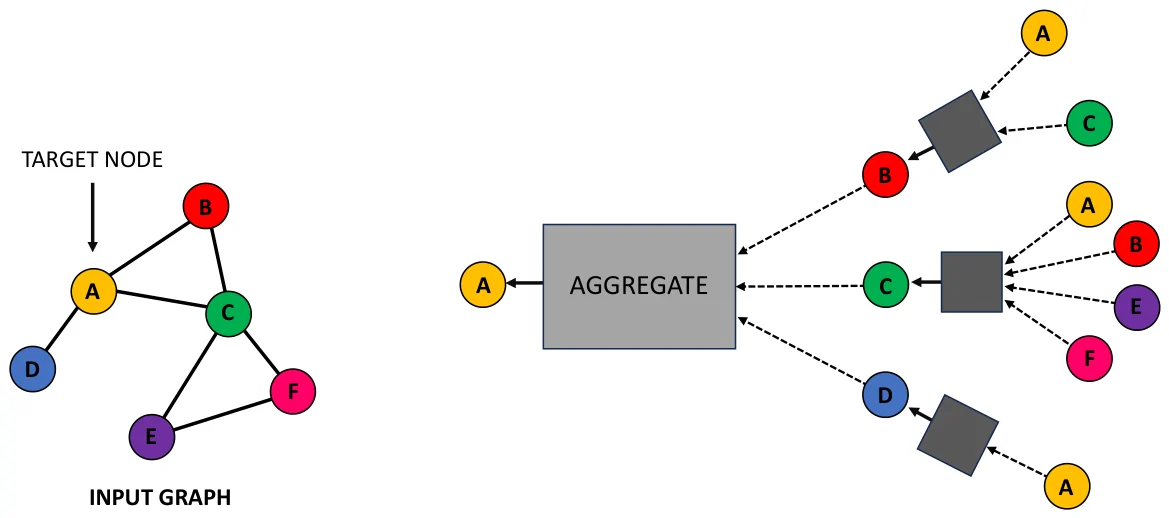
The message-passing mechanism in GNNs. The target node (A) collects information from its neighbors, who may first process their own neighborhood features before aggregation. The aggregated message is then used to update the embedding of node A. (Source: adapted from [21].)

**Figure 3:**
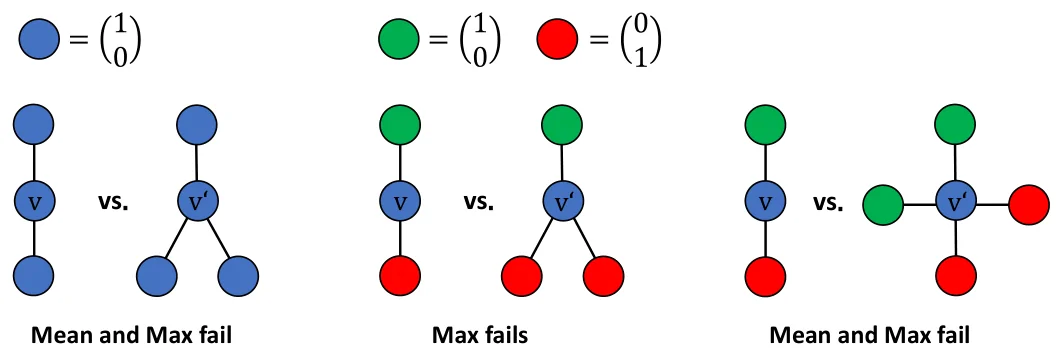
Examples of graph structures that mean and max aggregators fail to distinguish. As *mean* or *max* fail to distinguish graphs that differ only in neighborhood cardinality or composition, v and $v^{\prime }$ get the same embedding even though their corresponding structures differ. Here, node colors denote different node features. (Source: adapted from [38].)

**Figure 4:**
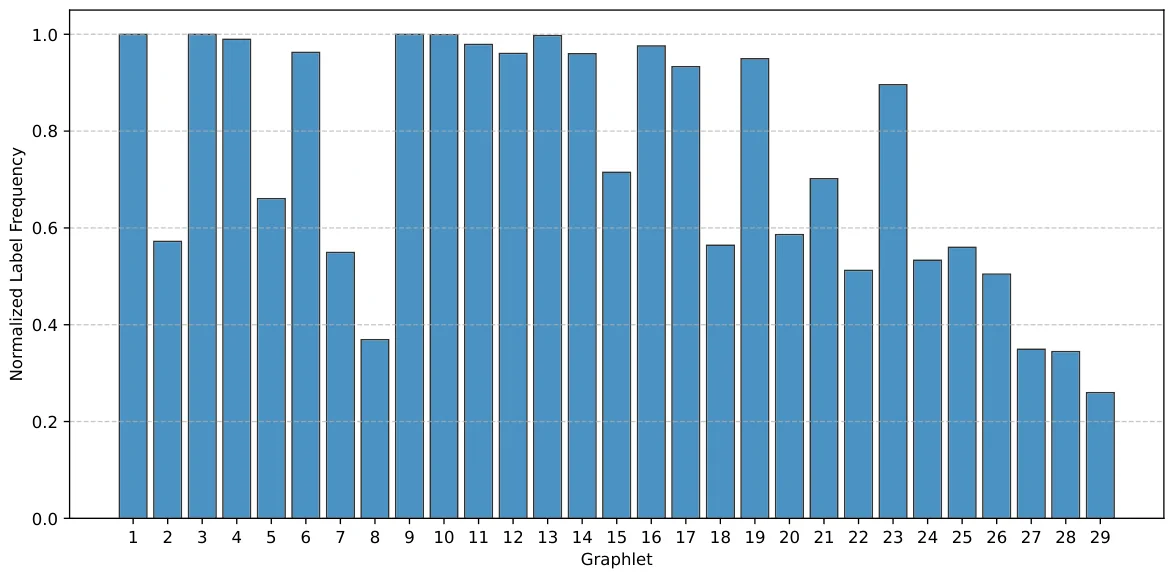
Normalized label frequency of nodes participating in graphlets 1 to 29. Ground-truth labels are obtained by binarizing ORCA-derived graphlet degree vectors.

**Figure 5:**
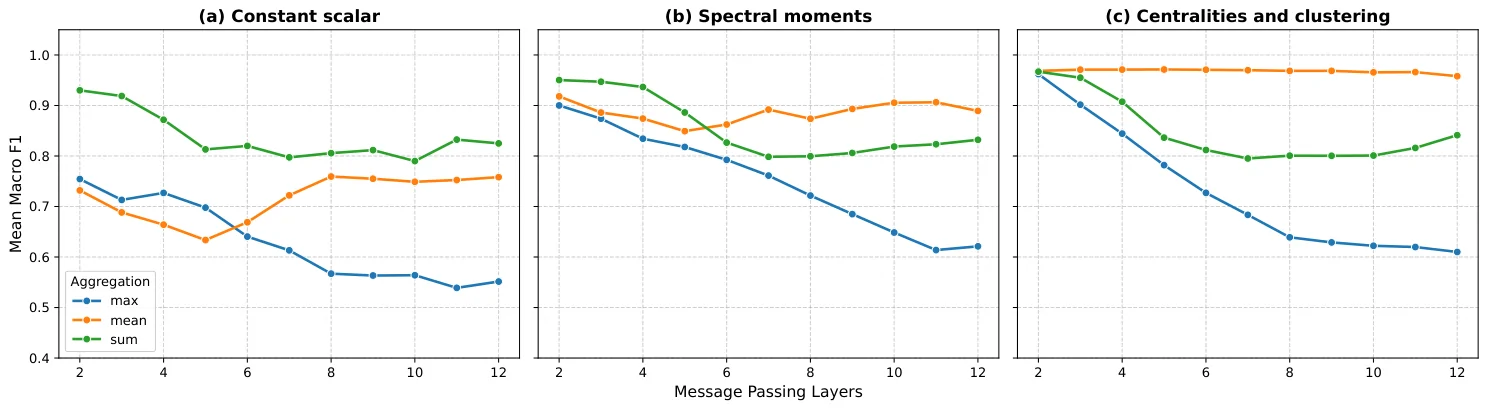
Effect of network depth and aggregation function across different feature sets.

**Figure 6:**
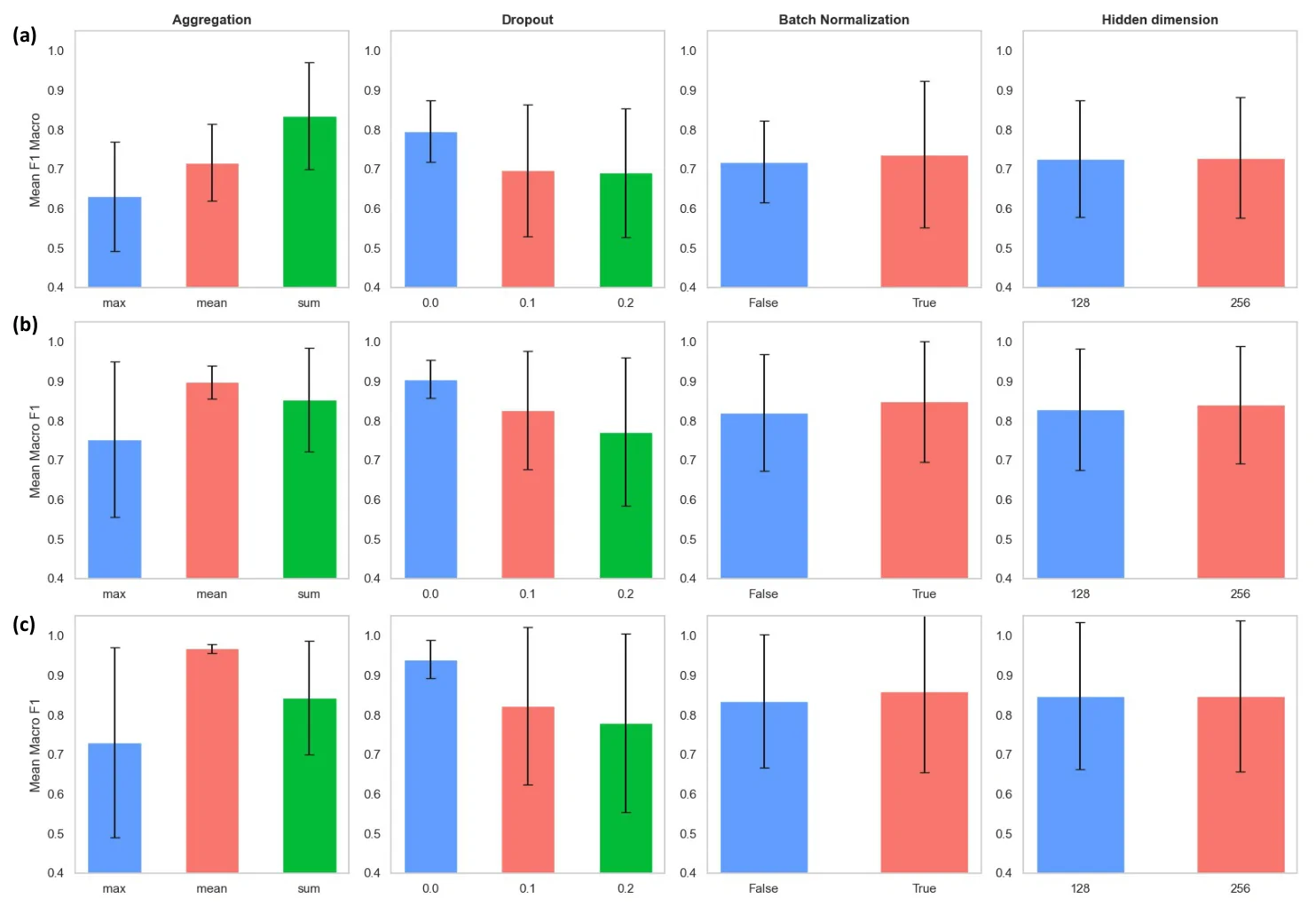
Mean macro F1 across different feature sets. Each subpanel reports the results of a full-grid search over architectural design dimensions: aggregation function, dropout rate, batch normalization, and hidden dimension size. Error bars represent standard deviations across all configurations sharing the same design choice. (**a**) constant scalar feature set, (**b**) spectral moments, (**c**) centralities and clustering coefficient.

**Figure 7:**
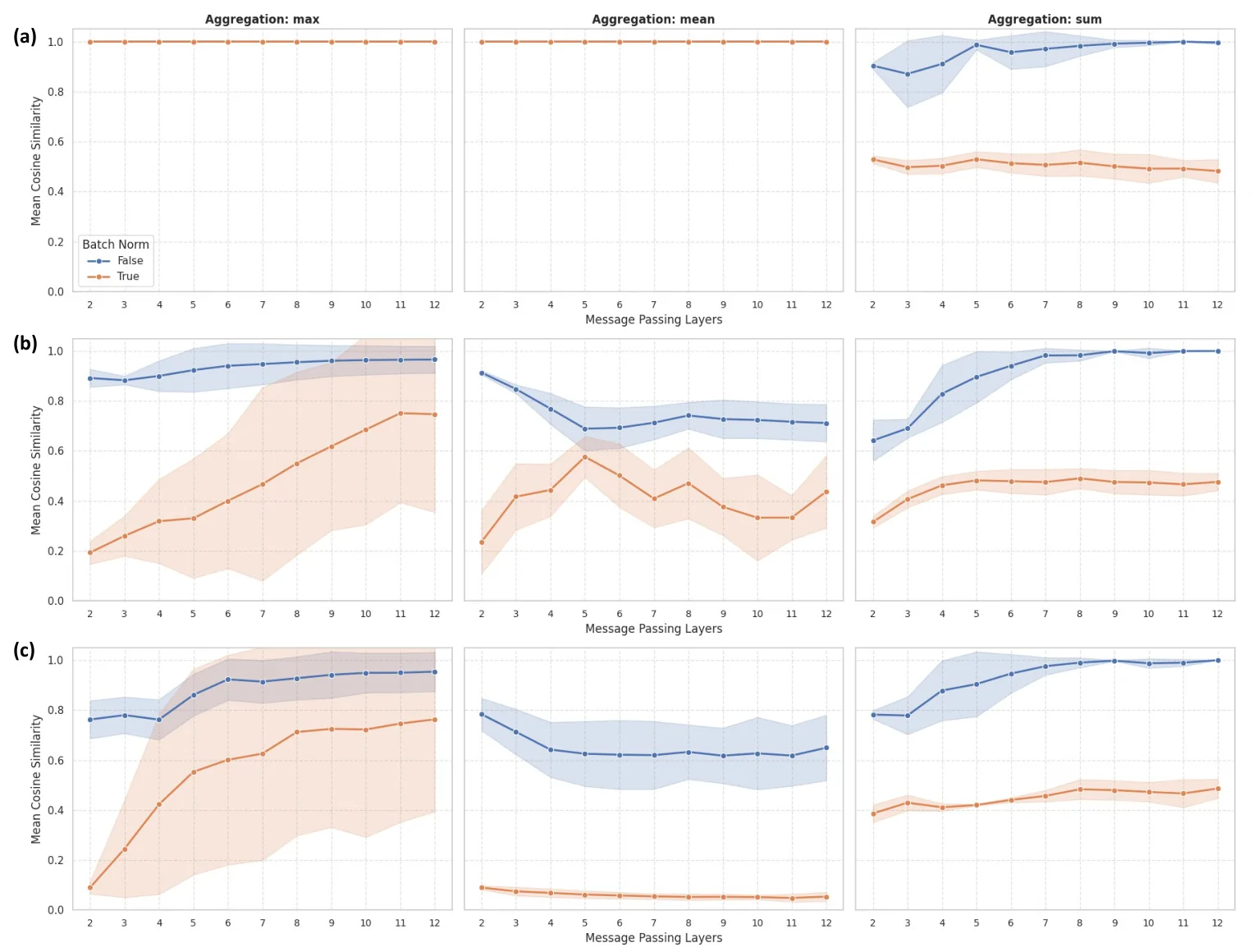
The mean cosine similarity of node embeddings across depths (2→12) showing the effect of Batch Norm on embedding separability. Shaded areas denote standard deviation across remaining design dimensions. (**a**) constant scalar, (**b**) spectral moments, (**c**) centralities and clustering coefficient.

**Figure 8:**
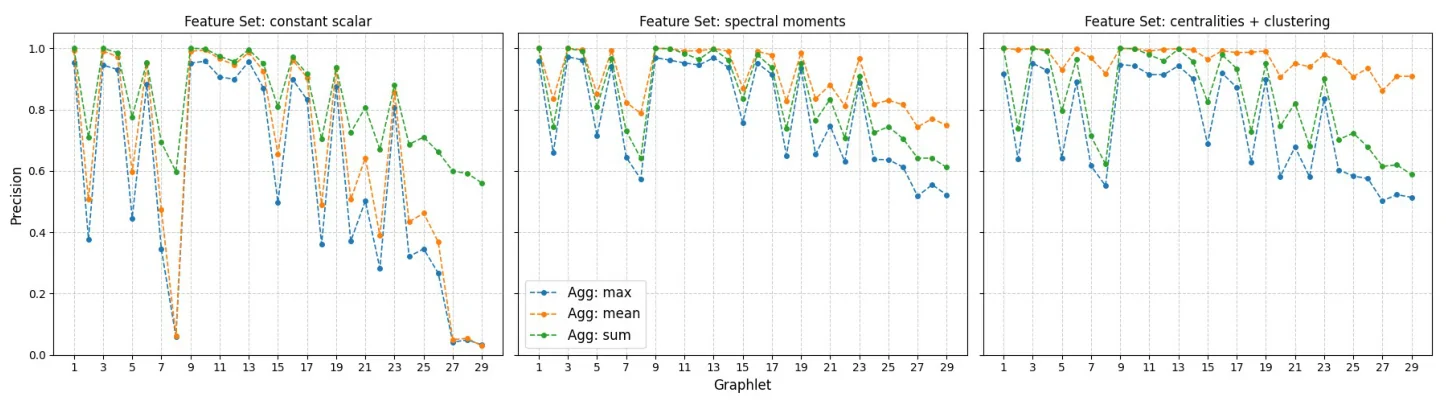
Mean graphlet precisions across 5 splits. The results highlight feature- and aggregation-dependent performance variations, reflecting both structural failure cases (e.g., in cycles and clique graphs) and class imbalance-driven underperformance in underrepresented graphlet classes.

**Figure 9:**
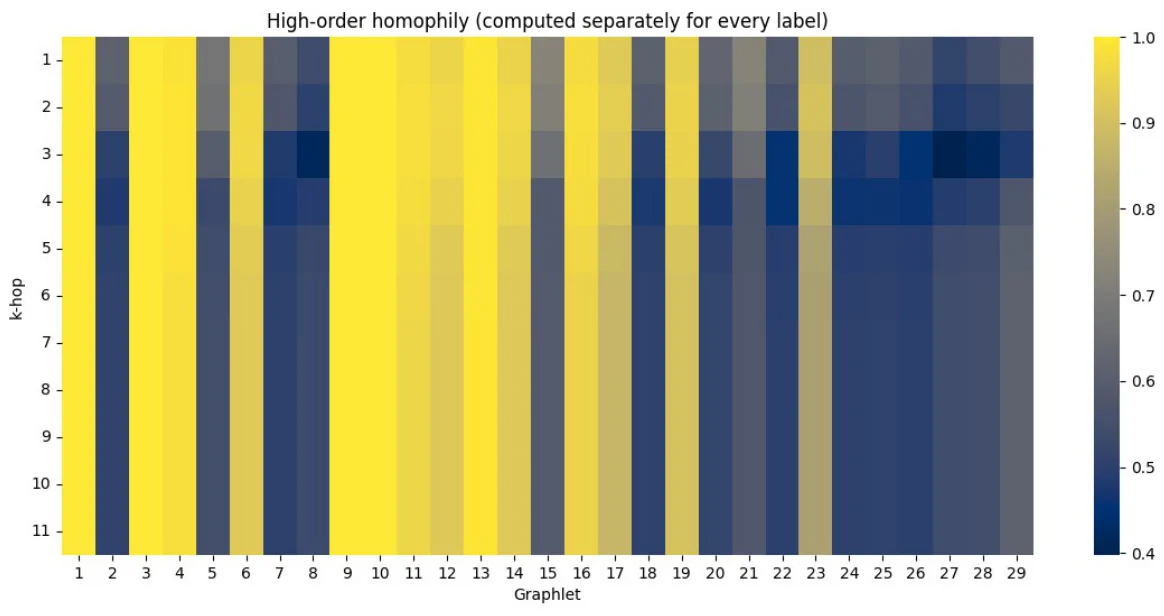
Higher-order label homophily per graphlet class across *k*-hop neighborhoods (k=1…11), computed via Equation (3). The values reflect the ratio of identically labeled neighbors at distance *k*.

**Figure 10:**
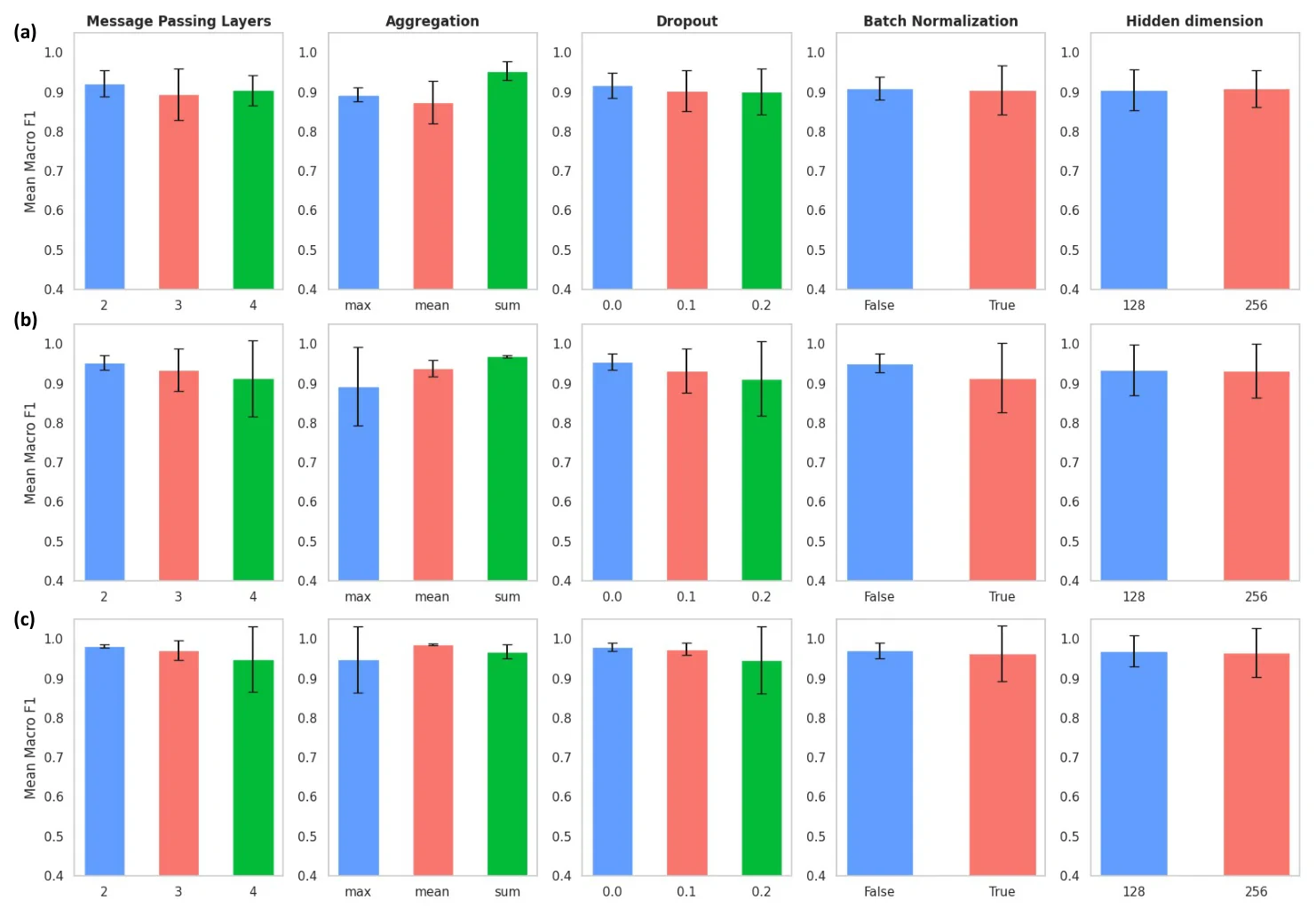
Mean macro F1 across different feature sets. Each subpanel reports the results of a full-grid search over architectural design dimensions: depth, aggregation function, dropout rate, batch normalization, and hidden dimension size. Error bars represent standard deviations across all configurations sharing the same design choice. (**a**) Constant scalar feature set, (**b**) spectral moments, (**c**) centralities and clustering coefficient.

**Figure 11:**
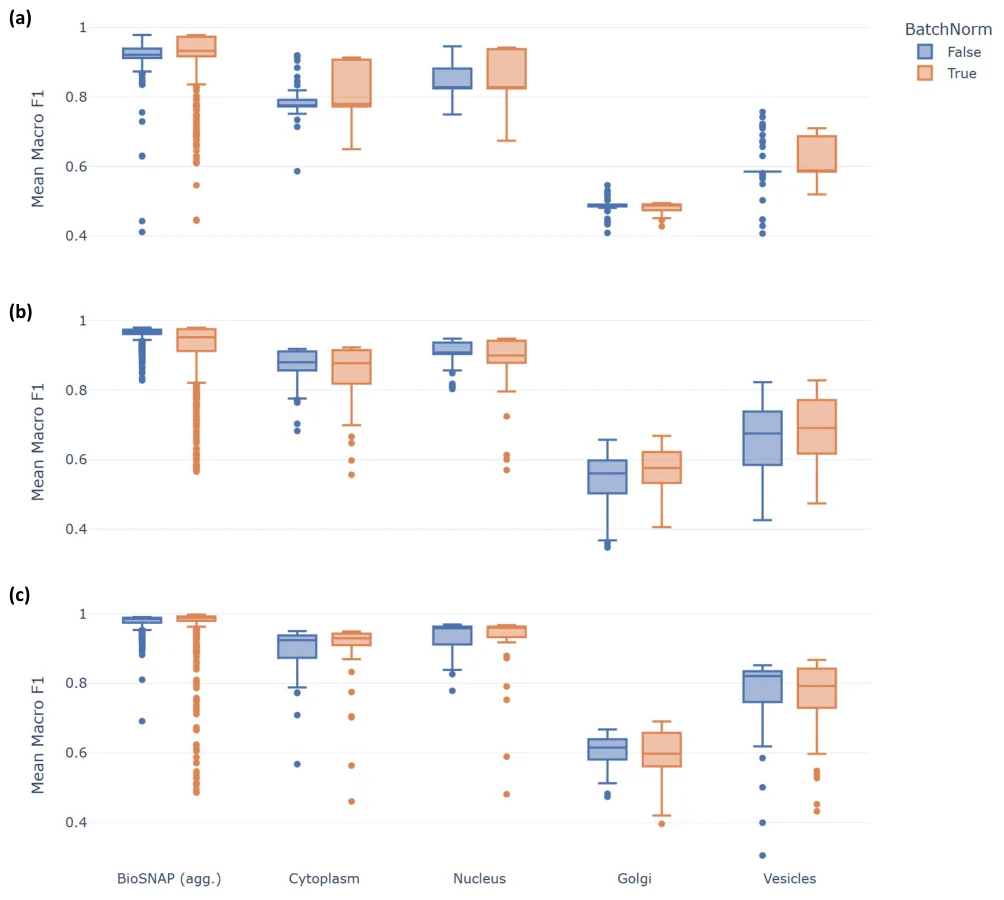
Mean macro F1 performance of inductive GNNs with and without BatchNorm. Mean macro F1 performance for configurations with (orange) and without (blue) BatchNorm across BioSNAP benchmarks and cell organelle graphs. Rows correspond to feature sets: (**a**) constant scalar, (**b**) spectral moments, and (**c**) centralities and clustering coefficients.

**Figure 12:**
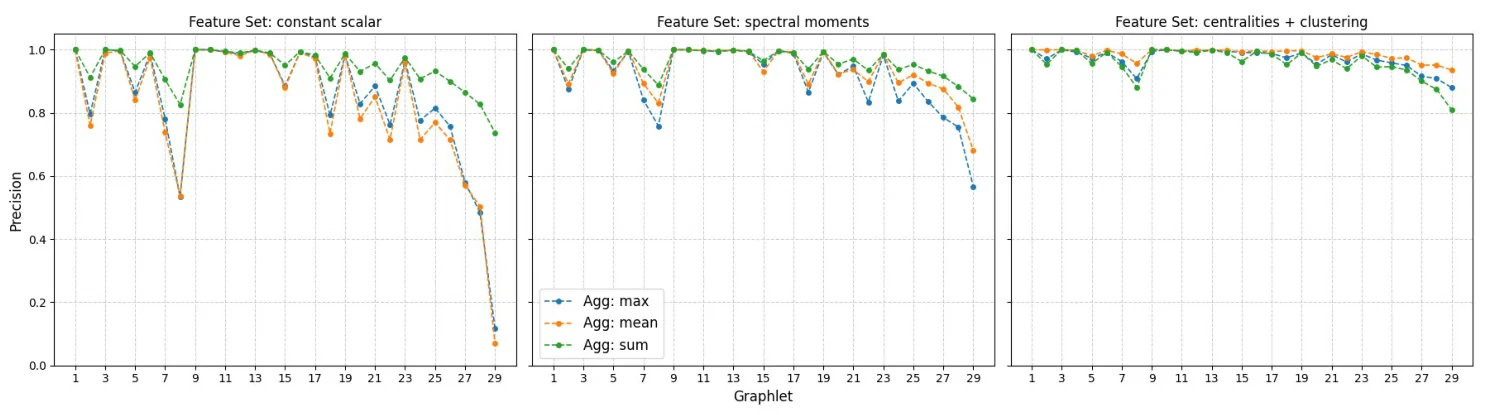
Mean graphlet precisions across 5 splits in the inductive setting. Compared to the transductive setup, the larger and more diverse training data yields smoother precision profiles, though Constant Scalar features still exhibit aggregation-dependent structural failure cases.

**Table 1:** Condensed configuration space for the inductive GNN setting.

Number of Layers	Aggregation	Batch Norm.	Dropout	Hidden Dim.
2, 3, 4	max, mean, sum	False, True	0.0, 0.1, 0.2	128, 256

**Table 2:** Macro F1 evaluation per feature set for inductive node classification. Comparison of feature sets across test graphs with associated homophily and Jensen–Shannon divergence (JSD).

Network	|V|	Homophily	JSD	Macro F1		
				Constant	Spectral	Node
BioSNAP						
1	3480	0.9756	$1.94\times 10^{-4}$	0.9751	0.9771	0.9771
2	3312	0.9787	$4.21\times 10^{-4}$	0.9780	0.9794	0.9794
3	3284	0.9765	$2.82\times 10^{-4}$	0.9764	0.9779	0.9779
4	3195	0.9762	$2.51\times 10^{-4}$	0.9753	0.9783	0.9783
5	3194	0.9776	$3.26\times 10^{-4}$	0.9772	0.9793	0.9793
6	3163	0.9751	$2.1\times 10^{-4}$	0.9749	0.9770	0.9770
9	2794	0.9773	$3.51\times 10^{-4}$	0.9774	0.9796	0.9796
10	2650	0.9762	$2.94\times 10^{-4}$	0.9765	0.9794	0.9794
11	2488	0.9753	$2.69\times 10^{-4}$	0.9769	0.9785	0.9785
13	2339	0.9754	$3.03\times 10^{-4}$	0.9753	0.9788	0.9788
14	2326	0.9712	$1.54\times 10^{-4}$	0.9757	0.9766	0.9766
15	2300	0.9734	$2.39\times 10^{-4}$	0.9758	0.9773	0.9773
17	1878	0.9717	$2.24\times 10^{-4}$	0.9736	0.9767	0.9767
20	1578	0.9677	$1.25\times 10^{-4}$	0.9715	0.9740	0.9740
21	1546	0.9640	$2.86\times 10^{-5}$	0.9679	0.9725	0.9725
22	1377	0.9679	$7.17\times 10^{-5}$	0.9707	0.9758	0.9758
23	1021	0.9605	$5.54\times 10^{-5}$	0.9594	0.9706	0.9706
Cell Organelles						
Golgi	192	0.8143	$1.22\times 10^{-1}$	0.4811	0.6568	0.6568
Vesicles	585	0.7946	$6.46\times 10^{-2}$	0.7098	0.8216	0.8216
Cytoplasm	3580	0.8995	$1.4\times 10^{-2}$	0.9149	0.9165	0.9165
Nucleus	4158	0.9371	$4.63\times 10^{-3}$	0.9432	0.9474	0.9474

Nodes (features) include node centralities and clustering coefficients.

**Table 3:** True counts (ORCA) vs. absolute differences for the probability-guided search under varying feature configurations. Nodes (features) include node centralities and clustering coefficients.

Graphlet	Total Count	Absolute Error		
		Constant	Spectral	Node
1	45,786,288	0	0	0
2	2,570,633	23	4	0
3	2,455,190,411	0	0	0
4	2,301,052,005	1	0	17
5	39,661,925	49	57	60
6	562,359,828	0	1	0
7	50,435,936	41	13	12
8	17,061,726	141	14	11

**Figure A1:**
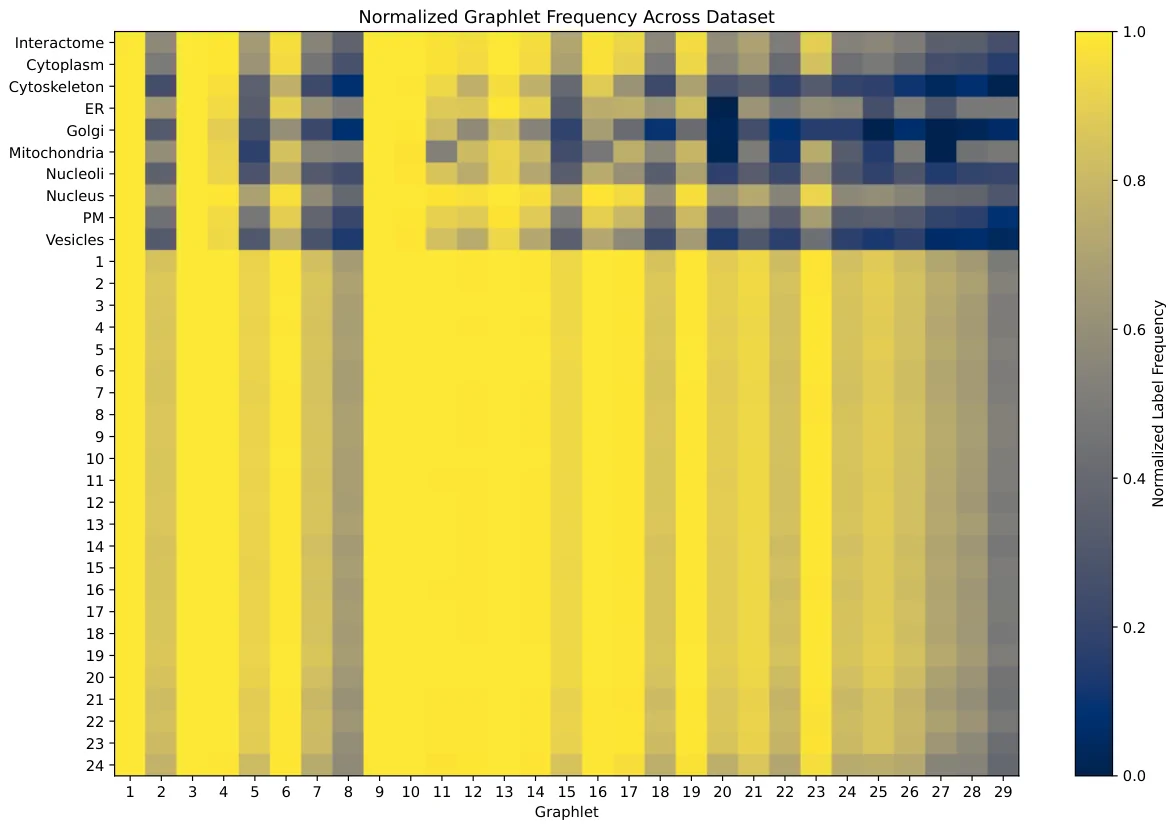
The normalized label frequency of nodes participating in graphlets 1 to 29 across the dataset. Ground-truth labels are obtained by binarizing ORCA-derived graphlet degree vectors. The interactome can be partitioned into nine subnetworks representing organelle-specific PPIs. The BioSNAP human PPI dataset contains 24 networks. (ER: Endoplasmic Reticulum. PM: Plasma Membrane.)

**Figure A2:**
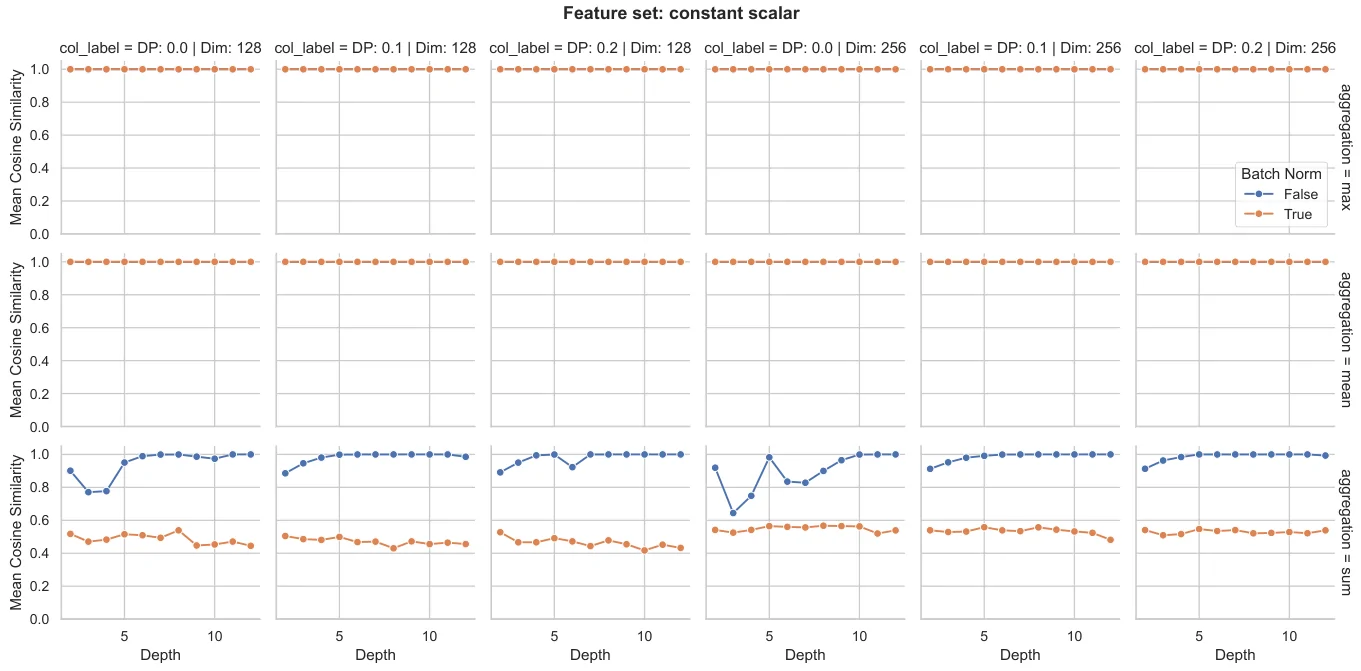
The mean cosine similarity of node embeddings across network depths (2→12) using a constant scalar as initial node features. Providing a fine-grained view of Figure 7, the layout contrasts aggregation operators, hidden dimensions (128 vs. 256), dropout rates (DP), and batch normalization (False vs. True). Higher similarity values at greater depths indicate severe embedding collapse due to oversmoothing. Because initial representations are identical across nodes, sum aggregation combined with batch normalization is crucial to preserve layer-wise feature separability and prevent immediate convergence to uniform embeddings.

**Figure A3:**
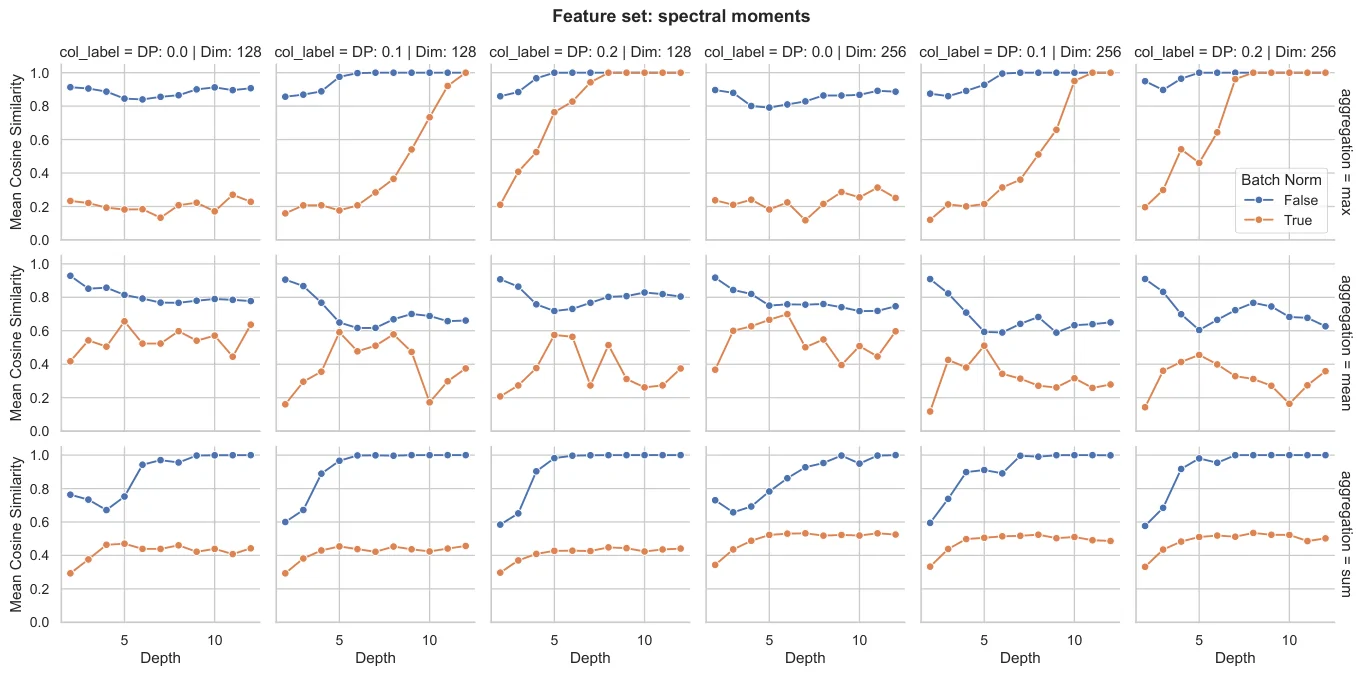
The mean cosine similarity of node embeddings across network depths (2→12) using spectral moments as initial node features. Providing a fine-grained view of Figure 7, the layout contrasts aggregation operators, hidden dimensions (128 vs. 256), dropout rates (DP), and batch normalization (False vs. True). Higher similarity values at greater depths indicate severe embedding collapse due to oversmoothing. Due to spatial overlap in 2-hop egonetworks, spectral moments exhibit higher initial similarity, rendering representations particularly sensitive to overmixing without batch normalization.

**Figure A4:**
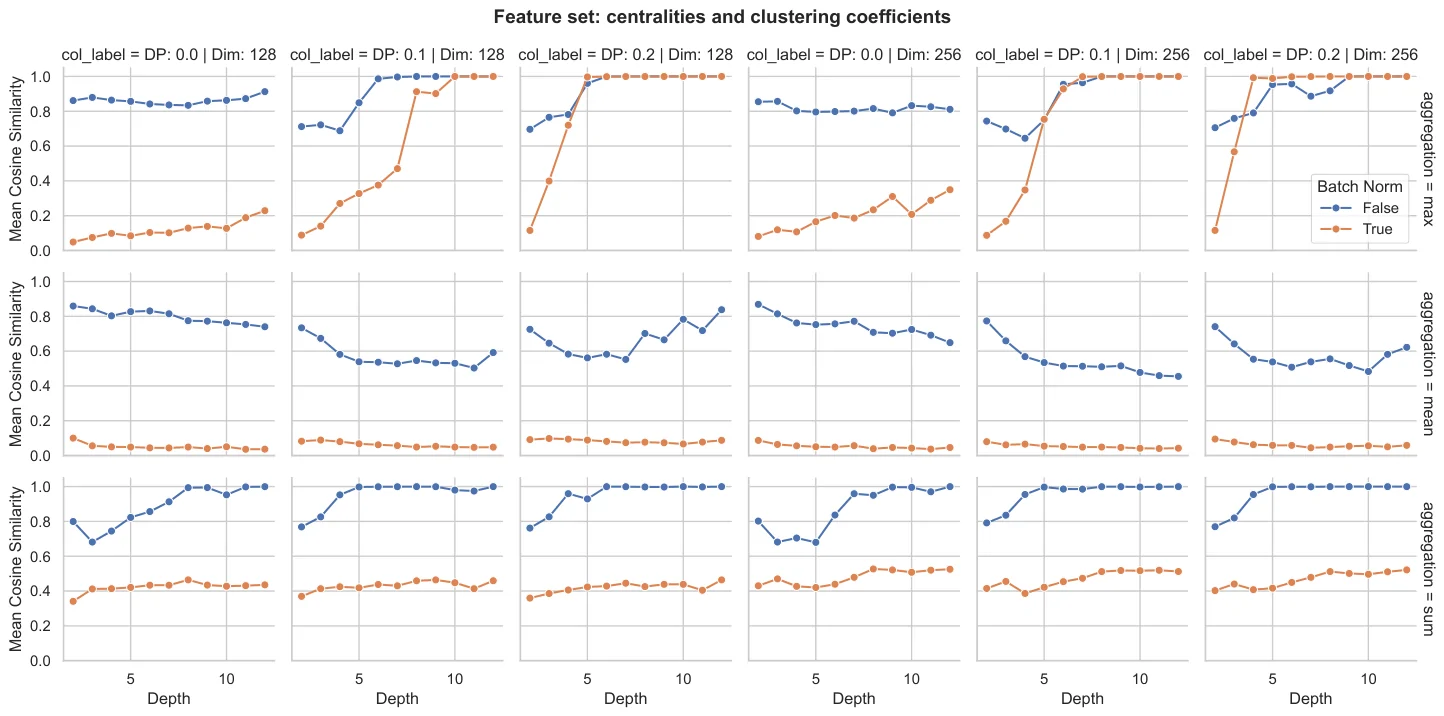
The mean cosine similarity of node embeddings across network depths (2→12) using centralities and clustering coefficients as initial node features. Providing a fine-grained view of Figure 7, the layout contrasts aggregation operators, hidden dimensions (128 vs. 256), dropout rates (DP), and batch normalization (False vs. True). Higher similarity values at greater depths indicate severe embedding collapse due to oversmoothing. Across configurations, batch normalization acts as a primary stabilizer against feature alignment, whereas applying dropout significantly accelerates the onset of oversmoothing.

**Figure A5:**
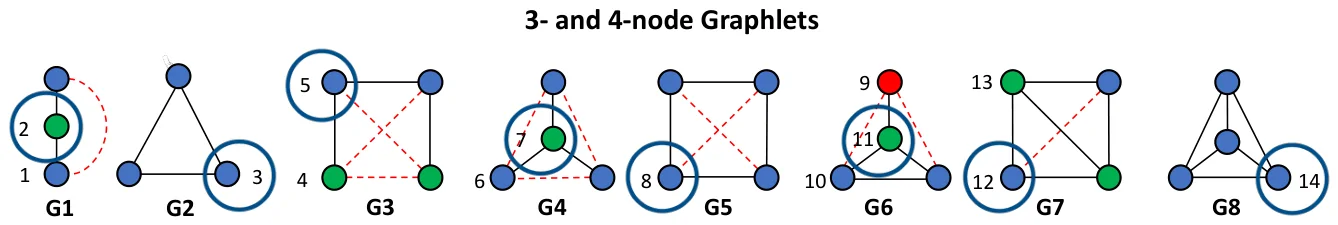
The heuristic detector workflow for 3- and 4-node graphlet enumeration. The circled orbit nodes serve as anchor points for the detector, activated when the model’s predicted probability exceeds 0.5 (p>0.5). From these anchor nodes, the detector scans immediate neighborhood structures for “deal-breaker” edges (highlighted in red dashed lines) whose presence would violate structural induction, thereby filtering out non-induced subgraph instances.

## Data Availability

Data are available in a publicly accessible repository. The interactome and subcellular localization data partitioned by cell organelles (enabling the generation of the organelle-specific graphs for the inductive setting) were obtained from the Supplementary Information of Thul et al. (2017) [7] (also deposited in the Human Protein Atlas at https://www.proteinatlas.org/humancell and https://www.proteinatlas.org/humanproteome/subcellular). The biomedical network datasets from BioSNAP (Stanford Biomedical Network Dataset Collection) can be found at https://snap.stanford.edu/biodata/ (accessed on 28 April 2025).

## Abbreviations

    The following abbreviations are used in this manuscript:

PPI (network)	Protein–Protein Interaction (Network)
GNN	Graph Neural Network
GCC	Greatest Connected Component
GDV	Graphlet Degree Vector
GDD	Graphlet Degree Distribution
JSD	Jensen–Shannon Divergence

## Appendix A. Dataset Description

**Table A1:** Dataset statistics and topological properties. Node and edge counts, diameter, and clustering coefficients across the dataset. Interactome can be partitioned into nine subnetworks representing organelle-specific PPIs. Inductive setting combines these 9 networks with 24 BioSNAP networks spanning diverse scales and structural characteristics. (ER: Endoplasmic Reticulum. PM: Plasma Membrane.)

Network	|V|	|E|	Avg. Degree	Diameter	Avg. CC
Interactome	7185	59,968	16.7	11	0.179
Cell Organelles					
Cytoplasm	3580	20,287	11.3	9	0.135
Cytoskeleton	1647	3391	4.1	11	0.087
ER	115	1341	23.3	9	0.523
Golgi	192	275	2.9	12	0.159
Mitochondria	241	1961	16.3	14	0.493
Nucleoli	418	1808	8.7	11	0.216
Nucleus	4158	36,504	17.6	9	0.198
PM	714	1847	5.2	11	0.145
Vesicles	585	1058	3.6	11	0.125
BioSNAP					
1	3480	53,377	30.7	8	0.173
2	3312	53,099	32.1	7	0.179
3	3284	50,737	30.9	6	0.179
4	3195	48,716	30.5	7	0.173
5	3194	50,322	31.5	6	0.178
6	3163	47,079	29.8	7	0.176
7	3021	45,669	30.2	7	0.174
8	2815	42,760	30.4	7	0.178
9	2794	42,659	30.5	7	0.176
10	2650	38,532	29.1	7	0.182
11	2488	35,195	28.3	7	0.183
12	2401	32,109	26.7	7	0.176
13	2339	32,715	27.8	7	0.186
14	2326	29,931	25.7	7	0.169
15	2300	30,664	26.7	7	0.182
16	2263	29,822	26.4	7	0.177
17	1878	23,134	24.6	6	0.187
18	1823	22,165	24.3	8	0.179
19	1819	22,884	25.2	7	0.184
20	1578	18,081	22.9	8	0.182
21	1546	16,153	20.9	8	0.182
22	1377	14,852	21.6	8	0.195
23	1021	9108	17.8	8	0.194
24	591	3854	13.1	8	0.229

## Appendix C. Supplementary Data on Subgraph Counting

**Table A2:** Graphlet frequencies across test graphs. Summary of exact graphlet frequencies across evaluated test graphs for 3-node and 4-node graphlets.

Network	G1	G2	G3	G4	G5	G6	G7	G8
BioSNAP								
1	4,435,654	205,243	268,142,903	265,135,531	4,136,734	58,081,764	4,868,244	758,069
2	4,549,154	205,112	274,536,842	293,250,632	4,424,211	61,426,078	5,063,773	689,276
3	4,183,375	196,969	249,401,320	245,409,230	3,972,468	54,958,440	4,696,293	719,133
4	3,837,477	181,772	224,981,404	203,450,229	3,592,526	48,315,024	4,107,243	634,256
5	4,158,481	199,340	249,157,012	239,442,133	4,072,717	55,287,211	4,769,207	724,058
6	3,632,729	171,533	208,670,884	192,154,143	3,277,716	44,885,664	3,823,518	572,250
9	3,250,347	155,083	182,245,477	162,058,536	3,074,656	40,100,297	3,490,926	455,258
10	2,850,625	135,875	151,656,063	144,389,616	2,549,695	34,187,219	3,019,601	376,833
11	2,447,654	119,443	125,707,639	109,956,789	2,154,089	28,179,563	2,522,302	311,825
13	2,203,210	110,337	109,511,281	94,516,099	1,909,096	24,945,900	2,273,777	289,512
14	1,923,112	81,279	90,329,023	82,019,757	1,493,161	18,889,665	1,566,902	135,033
15	2,003,290	88,990	95,825,551	84,919,150	1,629,625	20,463,191	1,746,201	163,632
17	1,342,671	63,425	56,784,359	49,101,895	1,010,347	12,874,269	1,140,128	106,259
20	920,718	46,742	34,819,371	27,873,576	634,800	7,795,340	726,092	84,278
21	794,022	36,841	28,500,494	24,190,960	509,081	627,0871	548,340	45,212
22	705,214	37,107	24,351,908	20,426,610	467,211	5,939,218	570,387	54,335
23	355,272	18,214	9,861,073	8,112,061	197,373	2,354,552	225,910	18,813
Cell Organelles								
Golgi	908	65	2138	1930	17	348	34	22
Vesicles	6323	275	33,141	23,562	271	4721	435	66
Cytoplasm	676,190	103,059	17,415,133	13,823,226	157,167	7,714,747	1,507,684	1,155,823
Nucleus	1,509,862	413,929	53,257,395	53,257,395	40,796,340	398,964	29,685,746	3,768,939

**Table A3:** **Constant Scalar:** Absolute error of graphlet counts across test graphs.

Network	G1	G2	G3	G4	G5	G6	G7	G8
BioSNAP								
1	0	0	0	0	6	0	0	0
2	0	1	0	0	0	0	2	2
3	0	0	0	0	0	0	0	1
4	0	0	0	0	1	0	0	4
5	0	0	0	0	0	0	0	19
6	0	0	0	0	0	0	0	18
9	0	0	0	0	0	0	2	3
10	0	0	0	0	0	0	0	3
11	0	0	0	0	0	0	0	0
13	0	0	0	0	0	0	0	0
14	0	0	0	0	3	0	0	0
15	0	0	0	0	1	0	0	0
17	0	1	0	0	0	0	0	1
20	0	1	0	0	0	0	0	7
21	0	5	0	0	1	0	0	16
22	0	1	0	0	0	0	1	6
23	0	0	0	0	0	0	0	6
Cell Organelles								
Golgi	0	4	0	0	13	0	20	7
Vesicles	0	3	0	1	14	0	11	25
Cytoplasm	0	1	0	0	7	0	4	14
Nucleus	0	6	0	0	3	0	1	9

**Table A4:** **Spectral Moments:** Absolute error of graphlet counts across test graphs.

Network	G1	G2	G3	G4	G5	G6	G7	G8
BioSNAP								
1	0	0	0	0	6	0	1	0
2	0	0	0	0	0	0	1	0
3	0	0	0	0	0	0	0	0
4	0	0	0	0	1	0	0	0
5	0	0	0	0	2	0	0	0
6	0	0	0	0	2	0	0	0
9	0	0	0	0	0	0	1	0
10	0	0	0	0	0	0	1	0
11	0	0	0	0	0	0	1	0
13	0	0	0	0	2	0	0	0
14	0	0	0	0	3	0	0	0
15	0	0	0	0	1	0	0	1
17	0	0	0	0	0	0	0	1
20	0	0	0	0	0	0	0	0
21	0	0	0	0	1	0	0	0
22	0	0	0	0	0	0	0	0
23	0	0	0	0	0	0	0	0
Cell Organelles								
Golgi	0	1	0	0	12	1	2	0
Vesicles	0	3	0	0	14	0	5	3
Cytoplasm	0	0	0	0	8	0	1	8
Nucleus	0	0	0	0	5	0	0	1

**Table A5:** **Centralities and Clustering Coefficient:** Absolute error of graphlet counts across test graphs.

Network	G1	G2	G3	G4	G5	G6	G7	G8
BioSNAP								
1	0	0	0	0	6	0	0	0
2	0	0	0	0	0	0	1	0
3	0	0	0	0	3	0	0	0
4	0	0	0	0	1	0	0	0
5	0	0	0	0	0	0	0	0
6	0	0	0	0	1	0	0	0
9	0	0	0	0	0	0	0	0
10	0	0	0	0	0	0	0	0
11	0	0	0	0	0	0	0	0
13	0	0	0	0	2	0	0	0
14	0	0	0	1	3	0	0	0
15	0	0	0	0	1	0	0	0
17	0	0	0	0	0	0	0	10
20	0	0	0	0	1	0	0	0
21	0	0	0	0	0	0	0	0
22	0	0	0	0	0	0	0	0
23	0	0	0	0	0	0	0	0
Cell Organelles								
Golgi	0	0	0	4	10	0	1	1
Vesicles	0	0	0	10	24	0	4	6
Cytoplasm	0	0	0	1	4	0	0	3
Nucleus	0	0	0	1	4	0	6	1
